# Establishing circularity: development and validation of the circular work value scale (CWVS)

**DOI:** 10.3389/fpsyg.2024.1296282

**Published:** 2024-04-05

**Authors:** Jannick Schneider, Clemens Striebing, Katharina Hochfeld, Timo Lorenz

**Affiliations:** ^1^Center for Responsible Research and Innovation, Fraunhofer Institute for Industrial Engineering, Berlin, Germany; ^2^Department of Psychology, MSB Medical School Berlin, Berlin, Germany

**Keywords:** work value, person–organization fit, measurement, validation, scale development, genetic algorithm, confirmatory factor analysis

## Abstract

**Objectives:**

Addressing the lack of German-language instruments, this study aims to develop a questionnaire that enables the measurement of work values. According to the theory of basic human values (Schwartz, 1992), a culturally fitting questionnaire is validated by covering constructs in the four broader dimensions of Social, Prestige, Intrinsic, and Extrinsic work values. Convergent, discriminant and incremental congruent validity are assessed.

**Method:**

Data were collected in a cross-sectional online-based panel survey. Individuals working more than 20 h per week were included (*N* = 1,049). Using a genetic algorithm, an economical and valid questionnaire was designed to assess work values.

**Results:**

The 11 work values are measurable with three items each. They provide a good fit to the data with support for strict measurement invariance. The empirical associations to estimate construct validity overall reflect expected relations to social and individualistic work motives, neuroticism, environmental awareness, and basic values. Furthermore, congruent incremental validity is supported with relations to value congruence of the person–organization fit, and multidimensional scaling supports the assumed theoretical circularity of the work values.

**Implications:**

This study developed a questionnaire that enables a theory-based valid measurement of work values. The questionnaire allows practitioners to economically collect information about the value structure of employees or applicants. Future research should consider the development of work values over time and investigate whether more distinctive constructs provide a better fit in the nomological network.

## 1 Introduction

Values play a significant role in the realm of organizations and work, attracting considerable interest from researchers and practitioners alike. For one, practitioners can be interested in capturing the value structure in personnel selection to identify individuals with the “right” values (Anglim et al., 2022). Values can provide insight into cultural fit, likelihood of quitting, and possibly even job performance. Furthermore, congruence between values and behavior is associated with well-being and reduced stress (Bojanowska et al., 2022). Research supports correlations to relevant outcome variables based on values (Glazer et al., 2004; Fischer and Smith, 2006; Anglim et al., 2022) such as attitudes toward diversity (Anglim et al., 2019), organizational commitment, and organizational citizenship behaviors (Arieli et al., 2020). Consequently, personal values have broad application possibilities for researchers and practitioners in personnel selection or organizational development.

To measure the value structure of employees or applicants, researchers and practitioners often rely on questionnaires that capture the general value structure (e.g., Schwartz, 2021). However, these questionnaires are intended to measure context-free universal value dimensions (Schwartz, 2016). The goal of these questionnaires is to capture values that apply in all situations across all domains of life. Thus, no specific value expressions are represented in the work context (Consiglio et al., 2017), and their use may disfavor applicant responses in selection settings due to low job references (Uggerslev et al., 2012). Hence, contextual item formulations are preferable in the work context due to their higher predictive and content validity (Shaffer and Postlethwaite, 2012; Moldzio et al., 2021; Potočnik et al., 2021; Sackett et al., 2022). Examples are contextualized traits like conscientiousness and emotional stability, yielding higher incremental validity above the non-contextualized counterparts. Against this background, this article seeks to develop and validate a contextualized questionnaire using a genetic algorithm to measure personal values represented in work contexts.

## 2 Literature review: from basic to contextualized work values

Values are cognitive representations of motives and secondary drivers of behavior (Schwartz, 1992, 2021; Kooij et al., 2011). These values play a significant role in influencing human action and decision-making as cross-situational goals that vary in importance (Arieli et al., 2020). The value structure of individuals and other social units function as guiding principles through which behavioral outcomes are evaluated as more or less desirable (Schwartz, 1994).

Schwartz’s (1992) theory of basic human values established a widely used and empirically supported theoretical framework that is applied across multiple areas of psychology (e.g., political attitudes and organizational contexts; Davidov et al., 2008; Sagiv and Schwartz, 2022). A total of 10 universal, cross-context values (see Table 1) have been supported in their discriminant, predictive, and factorial validity in samples from more than 80 countries (Schwartz, 1992, 1994; Schwartz and Boehnke, 2004; Schwartz et al., 2012). The 10 values can be expanded to a refined theory of basic values with 19 more narrowly defined constructs that represent the motivational continuum in a more distinctive way and specify the broader dimensions (Schwartz et al., 2012). The theoretical values represent two pairs of higher value dimensions with contrasting motivational bases and desirable goals (Schwartz, 2021): Openness to Change (importance of independent thinking, acting, feeling, challenging, and changing) contrasted with Conservation (importance of self-retention, securing the past, order, and resistance to change), Self-Enhancement (importance of one’s own interests, relative success and dominance over others) in contrast to Self-Transcendence (concern for the well-being and interests of others).

**Table 1:** Definitions of work values and corresponding basic values.

Work value	Work value definition	Basic value	Value-Dimension
Authority	Social status and prestige in the work setting are expressed through leadership roles and influence.	Power	Self-Enhancement/Prestige
Ambition	Personal success at work is defined by recognition of one’s abilities and products in the organization.	Achievement	
Enjoyment	Pleasure in doing work, compatibility between work and one’s recreational and leisure interests	Hedonism	Openness to Change/ Intrinsic
Variety	Variety, novelty, and challenges in work situations and contexts	Stimulation	
Autonomy	Independent thought and decision-making, creating, and exploring at work; freedom to choose how to perform one’s job	Self-Direction	
Social Justice	Fairness, respect, and protection against discrimination for all members of the work organization; socially responsible policies	Universalism	Self-Transcendence/Social
Environmental Sustainability	Protecting nature, pursuing sustainable actions at work, ensuring ecological well-being of the environment	Universalism	
Helping and Supporting	Devoting oneself to the needs of people with whom one is in frequent work contact and creating harmonious and supportive work relationships	Benevolence	
Rule Respecting	Complying and adapting to management expectations and norms, sacrificing personal inclinations to preserve organizational order	Conformity	Conservation/Extrinsic
Traditional Values	Respect, acceptance, and diffusion of organizational traditions, culture, and customs at work	Tradition	
Safety	Safety, stability, health, avoiding risks in the work and organizational setting	Security	

Labels based on Albrecht et al. (2020); Definitions based on Consiglio et al. (2017) and Albrecht et al. (2020).

According to Schwartz (2021), values form a coherent structure in a circular arrangement. The values line up according to the compatibility or conflict potential of the underlying goals and higher value dimensions. Thus, values with conflicting goals are further apart in the circular arrangement than values representing more compatible goal states (Schwartz, 1992, 2021). This assumes that compatible values guide similar perceptions, preferences, and behaviors (underlying goals are more likely to be pursued in similar actions). In contrast, when values conflict, the pursuit of one goal opposes the pursuit of another goal (Schwartz, 1992, 2021; Maio et al., 2009).

De Clercq et al. (2008) argued that the theory of basic human values should be applied to the work context for consideration of employees’ values. The theory provides a universal, cross-cultural context that relates individual values at work to each other and can cluster individual and organizational values. Ros et al. (1999) described work values as the specific expression of basic values in the work context. Like basic values, they are guiding principles which are hierarchically ordered according to their importance. Furthermore, they reflect the subjective importance of work-related goals and describe what a person expects from his or her work.

The four dimensions of basic values (Self-Transcendence, Self-Enhancement, Openness to Change, and Conservation) reflect Social, Prestige, Intrinsic, and Extrinsic work values in the circular arrangement in work contexts (Ros et al., 1999; Johnson, 2001; Krumm et al., 2013; Borg et al., 2019; Sulistiobudi and Hutabarat, 2022). Social work values reflect the meaningfulness of positive social relationships and the possibility to contribute to society. Prestige-related work values represent goals regarding power, authority, influence, and success at work. Autonomy, enjoyment, and creativity are expressions of Intrinsic work values. In contrast, Extrinsic work values relate to job security and maintaining order in an employee’s life. The definitions of the basic values applied to the work context are shown in Table 1.

Albrecht et al. (2020) supported the extension of the work values by an 11th construct. Based on Exploratory Factor Analysis, they identified the value of Environmental Sustainability (subsumed under Universalism in the theory of basic human values; Schmidt et al., 2007) as an independent dimension. Here, the increasing subjective significance of aspects such as corporate social responsibility and environmental protection in work contexts is evident (Albrecht et al., 2020). Additionally, Albrecht et al. (2020) understood Safety as the importance of safety climate (interpersonal), which subordinates the value under the dimension of Self-Transcendence/Social. Nonetheless, Schwartz et al. (2012) emphasize that Safety can be considered both self-related and social. Simultaneously, the definition, according to Consiglio et al. (2017), contains further aspects beyond the physical safety climate. This will be considered in the following section.

### 2.1 Measurement of contextualized work values

As mentioned above, to measure values in a work-related manner, appropriate questionnaires with contextual relevance and high predictive validity are needed. These questionnaires should reflect the theory of basic human values in the work context. To our knowledge, there are no questionnaires available in the German work context that replicate and validate the factor structure of Schwartz’s basic values (2021).

#### 2.1.1 German-language questionnaires

The German adaptation of the Super Work Value Measure (Seifert and Bergmann, 1983) is not aligned with the theory of basic human values and is outdated. With their Munster Work Value Measure, Krumm et al. (2013) captured single items per value and not the 10 broader value constructs. They extend the basic values by 11 additional ones, including values that are especially relevant to older workers. Stiglbauer et al. (2022) developed a questionnaire to assess work orientations partially based on individual’s values in the context of employer branding. They integrate various aspects of the meaning of work under generational differences. The relevant circularity of the theory of basic human values is not studied in the work context, and various theoretical assumptions are integrated in their approach. To some extent, the questionnaire focusses more generally on the expectations of an employer and not on the work itself. Moreover, there are variations in values across cultures and diverse intercultural perspectives on work (Shi et al., 2023). Conducting an initial assessment to ascertain the applicability and validity of Schwartz’s theory of basic human values within the context of German workplaces is imperative.

#### 2.1.2 Questionnaires in other cultural contexts

Outside the German context, various questionnaires do not adequately represent the theory of basic human values or fail to replicate its proposed factor structure in work contexts (Arciniega and González, 2000; Avallone et al., 2010; Busque-Carrier et al., 2022). This is critical because Schwartz’s theory serves as a broad theoretical framework which specifies the interrelations of universal values. The broad value conceptualizations are essential to differentiate between distinct motivational bases and can be used to adequately aggregate work value items from a wide variety of work value lists. Therefore, the theory provides a valid foundation for researching work values under the premise of replicating the motivational continuum in work contexts (De Clercq et al., 2008). Consiglio et al. (2017) were able to support the application of universal values in the work context for the first time using ranking scales. More recently, Albrecht et al. (2020) extended the use of the questionnaire by Consiglio et al. (2017) with rating scales. This has the advantage of being able to compare longer lists of values, and participants do not have to discriminate between the importance of individual values (Alwin and Krosnick, 1985; Schwartz, 1994; Sagiv and Schwartz, 2022).

### 2.2 Aim of this study

In the present study, a German work value questionnaire will be developed and validated based on the Values at Work Scale from Albrecht et al. (2020). We pursue the following objectives.

#### 2.2.1 Translation, adaptation, and advancement of the Values at Work Scale

We want to adapt the questionnaire from Albrecht et al. (2020) and develop a culturally fitting questionnaire with accurate validation procedures (MacKenzie et al., 2011; International Test Commission, 2017; Boateng et al., 2018). In particular, the consideration of content validity and the cultural fit of the item formulation plays a significant role. Moreover, the value of Environmental Sustainability needs to be operationalized more comprehensively for higher measurement accuracy. In the initial version, the value of Safety should include items for both intrapersonal and interpersonal Safety to meet theoretical propositions (Schwartz et al., 2012; Consiglio et al., 2017).

#### 2.2.2 Construct validity

To evaluate factorial validity, the questionnaire will be generated as economically as possible and with a good fit to the training data based on a genetic algorithm (Schultze, 2017). As a result, we expect a good fit of the measurement model to the test data (H1) in Confirmatory Factor Analysis (CFA). Building on the theoretical circular arrangement of Schwartz’ values, we expect a continuous circular pattern (H2) comparable to Schwartz et al. (2001, 2012), Albrecht et al. (2020) and Borg et al. (2019) using Multidimensional Scaling (MDS).

Albrecht et al. (2020) and Consiglio et al. (2017) have advocated the expansion of construct diversity to capture the nomological validity of work values. Therefore, to assess convergent validity, correlations to specific motives will be considered within the circular array of values. We expect social work motives to correlate more positively with Social work values than with Prestige work values (H3a). We further hypothesize that individualistic work motives correlate more positively with Intrinsic work values than with Extrinsic work values (H3b). To test the convergent validity of the additionally posited value of Environmental Sustainability, the correlation to environmental awareness will be assessed. Here, we expect a positive correlation (H4).

We address discriminant validity and whether the corresponding questionnaires of the construct are empirically distinct (Rönkkö and Cho, 2022). It will be assessed in relation to neuroticism as an affective-oriented trait with personal tendencies to negative mood states (McCrae and Costa, 2003). Data from a comprehensive meta-analysis support the notion that this affective trait has no or only very small correlations with cognitive-based basic values (Parks-Leduc et al., 2015). Since values tend not to have direct implications for stress (Roccas et al., 2002; Sagiv et al., 2004), we also expect a sufficient empirical distinction of work values to neuroticism in our study (H5).

#### 2.2.3 Criterion validity

To establish correlations with relevant outcome variables, work values will be considered in relation to value congruence in the person–organization fit (PO-Fit; Kristof, 1996; Cable and DeRue, 2002). The perceived complementary fit of one’s own values to organizational values emerges as an important variable in organizational settings (for relevant correlations, see Kristof-Brown et al., 2005, 2023; Arthur et al., 2006; Uggerslev et al., 2012; Etzel and Nagy, 2020; Straatmann et al., 2020; Ghielen et al., 2021). Specifically, we capture the congruent incremental validity of work values versus Schwartz’s (1994) basic values. Because work values represent a contextualized conceptualization, we expect a significant increase in explained variance compared to the four value dimensions of basic values (H6).

## 3 Method

We report how our sample size was determined, the basis on which cases are excluded, and all manipulations and measurement tools (Simmons et al., 2011; Kline, 2019, p. 64). We used a checklist to ensure transparency in our research (Aczel et al., 2020; see Appendix 1) and preregistered the study.^1^ To develop a valid and short questionnaire for a useful application in organizational and research settings, we conducted a quantitative representative cross-sectional online panel survey in June 2023.

### 3.1 Sample

The addressed population is composed of working-age individuals (18–69) in Germany with at least 20 working hours/week to establish a sufficiently large reference to work activity. Based on different recommendations for CFA sample sizes (e.g., *N:q*-ratio, *N*_minimum_ = 200; Gnambs, 2013; Kline, 2016; Muthén and Muthén, 2017; Wang and Rhemtulla, 2021; Nye, 2022; R-package: semTools) and the need for a randomized split of the data set, as well as a buffer for potential case exclusion, a minimum of *N* = 990 was set (*n*_training_ = 450, *n*_test_ = 350, *n*_buffer_ = 190). The online panel sample was based on representative distributions concerning age and gender for Germany (study incentive: 2,50€). The final sample was acquired via e-mail invitations of panelists [*N* = 1,048; 46.66% women, 7.44% with migration background,^2^ 29.7% with management responsibilities, *M_tenure(years)_* = 12.43, *SD_tenure(years)_* = 10.78, *M_age_* = 44.14, *SD_age_* = 12.43, *M_workinghours/week_* = 37.64, *SD_workinghours/week_* = 6.75]. The distribution of industrial sectors and educational levels is presented in Tables 2 and 3. To ensure the quality of the panel sample, we referred our survey design to established best practices (Porter et al., 2019; Aguinis et al., 2021; Ward and Meade, 2023).^3^

**Table 2:** Distribution of industrial sectors (*N* = 1,048).

Industrial sector	Construction	Consulting	Education	Energy and water supply	Research and development	Health and social work	Real estate and housing	Trade, industry, credit and insurance	Information technology and communication	Public administration	Tourism and leisure	Manufacturing	Transport and logistics	Other
*N* %	5	4	6	2	3	12	1	11	10	11	3	8	7	16

**Table 3:** Distribution of educational level (*N* = 1,048).

*Education*	Secondary school certificate	General qualification for university entrance	Apprenticeship	Bachelor Degree	Master Degree	Doctorate Degree	Habilitation
*N* %	12	12	37	15	20	3	0.1

### 3.2 Materials

#### 3.2.1 Circular Work Value Scale

We translated the Values at Work Scale (Albrecht et al., 2020) into German following the guidelines of the International Test Commission (2017). The questionnaire underwent back-translation by two native English speakers with relevant cultural backgrounds. This process ensured an accurate reflection of the questionnaire’s meaning. Additional items were included for each work value dimension to facilitate algorithm-based item selection (ABIS) and to address all theoretical facets. Environmental Sustainability and Safety were specifically addressed with items more aligned with theoretical propositions. Construct definitions (Table 1) and existing German-language questionnaires for assessing work values (Seifert and Bergmann, 1983; Krumm et al., 2013; ISSP Research Group, 2017) guided the formulation of items. Selection was based on content validity. The questionnaire initially consisted of 77 items (7 per work value dimension) using a 7-point Likert scale (1 = “completely unimportant” to 7 = “very important”). To ensure content validity, five expert interviews were conducted with researchers from work and organizational psychology. These were followed by eight cognitive interviews with individuals from the target population^4^ (MacKenzie et al., 2011; Boateng et al., 2018). The received feedback led to iterative adjustments in wording and cultural appropriateness,^5^ resulting in the final set of items presented in Appendix A2.

#### 3.2.2 Measures to test convergent and discriminant validity

To evaluate convergent validity, the Inventory for the Assessment of Work Motivation—Short Form (IEA-K; Kanning, 2016) was selected for relations with work motives. The extended version shows satisfying psychometric quality (Lang et al., 2018). For the correlations in the circular arrangement of the work values, the scale on individualistic motives (12 items, e.g., “take responsibility myself”; *α* = 0.89; *ω* = 0.92) and on social motives (six items, e.g., “exchange information with colleagues also about private matters,” *α* = 0.79; *ω* = 0.88) was selected. Answer scales ranged from 1 = “unimportant for me” to 5 = “extremely important for me.”

For correlations with the newly set up work value of Environmental Sustainability, five items of the German version of the New Ecological Paradigm questionnaire on environmental awareness (Schleyer-Lindenmann et al., 2018; α = 0.85; ω = 0.87) were included (e.g., “If things continue on their present course, we will soon experience a major ecological catastrophe.”; 1 = “do not agree at all” to 6 = “completely agree”). Discriminant validity is to be tested by correlations with the Big Five-dimension neuroticism. The German version of the Big Five Inventory Short (three items, BFI-S; Schupp and Gerlitz, 2014, e.g., “I am someone who often worries.”; α = 0.82; ω = 0.83; 1 = “not applying at all” to 7 = “fully applies”) was used.

#### 3.2.3 Measures to test incremental congruent validity

The PO-Fit criterion in the incremental congruent validity test is assessed via three items from Cable and DeRue (2002) on value congruence (e.g., “The things that I value in life are very similar to the things that my organization values”; *α* = 0.93; *ω* = 0.93; 1 = “do not agree at all” to 7 = “completely agree”). Incremental validity is to be assessed against each of the four basic value dimensions of the Higher-Order Value Scale-17 (Lechner et al., 2022). Here, the four dimensions (Openness to Change, e.g., “It is important to her/him to develop her/his own opinions,” *α* = 0.83; *ω* = 0.85; Conservation, e.g., “It is important to her/him to maintain traditional values and ways of thinking,” *α* = 0.66; *ω* = 67; Self-Enhancement, e.g., “It is important to her/him to show that her/his performance is better compared to the performance of other people,” *α* = 0.74; *ω* = 0.76; Self-Transcendence, e.g., “It is important to her/him to help the people dear to her/him,” *α* = 0.82; *ω* = 0.85; 1 = “is not at all similar to me” to 6 = “is very similar to me”) are assessed using 17 items.

Furthermore, based on empirical evidence, different control variables were integrated. The age, gender, and tenure of respondents have been shown to impact basic and work values (Consiglio et al., 2017). We conducted a pre-test of the final online questionnaire with 13 participants to check the comprehensibility and questionnaire design. All items were presented in a forced choice format to ensure data completeness. The survey was administered in German.

### 3.3 Statistical analysis

Careless or inattentive response patterns and outliers were analyzed by multiple mechanisms (Schroeders et al., 2022; Ward and Meade, 2023). We used autocorrelation screening (R package: responsePatterns; Gottfried et al., 2022), long string analysis, intra-individual response variability and mahalanobis distance (R-package: careless; Curran, 2016). Conspicuous responses were further investigated. We excluded 195 data entries based on the applied techniques and short response times on individual pages. The final sample size included 853 participants.

Due to current debates on validity concerns of measures in psychological science (Flake et al., 2017; Hussey and Hughes, 2020; Shaw et al., 2020) and the need for short questionnaires (Fuchs and Diamantopoulos, 2009), the data will be analyzed with ABIS using a genetic algorithm. In the field of psychological assessment, algorithms are increasingly used in item selection and questionnaire development (Algner and Lorenz, 2022; Kerber et al., 2022; Pundt et al., 2022). Compared to classical approaches, algorithms have the advantage of being more objective and efficient with respect to defined criteria to find a (nearly) finite solution (Leite et al., 2008; Olaru et al., 2015). Empirical studies suggest that the use of algorithms leads to similar or better results in scale construction than traditional approaches (Sandy et al., 2014; Schroeders et al., 2016; Olaru and Danner, 2021). However, the need for a rigorous theory-driven item development covering all construct-specific properties must be considered (Dörendahl and Greiff, 2020).

The goal of ABIS is to select those items from an initial item pool that fulfill defined criteria (e.g., the best representation of the construct or best fit to the data). In this context, the selection of items and the development of an economic questionnaire can be defined as a combinatorial problem (Schroeders et al., 2016; Kerber et al., 2022). Based on a given set of items (here, 77 items), a questionnaire with 33 items should be developed with satisfying quality. Thus, the computation of the single best solution would be disproportionately time-consuming with average computational power (possible combinations: 96.549.157.373.046.880). Meta-heuristic like genetic algorithms are utilized to handle the complexities of such combinatorial optimization problems. Genetic algorithms are based on principles of natural selection (Holland, 1992; Schroeders et al., 2016). Since this is a meta-heuristic and estimation-based approach (Blum and Roli, 2003), it is not a procedure to find the single best solution (Yarkoni, 2010). However, the benefit is to increase the psychometric quality of the whole questionnaire under high efficiency (low demand of time and computational power; Dörendahl and Greiff, 2020) and the consideration of diverse item combinations (Olaru and Danner, 2021). The advantage of genetic algorithms is that items are not considered in isolation. Item quality is always evaluated considering specified criteria against the background of the entire questionnaire in CFAs (Schultze, 2017).

With a genetic algorithm (R-package: stuart), the initial collection of 77 items is to be reduced based on evolutionary selection processes with the goal of an optimal or near-optimal solution. The basis for the survival of an item in the item pool is its quality (called “fitness”; Galán et al., 2013). The algorithm is based on two processes: Variation (recombination and mutation) and selection. Variation promotes diversity and novelty of items, whereas selection rewards quality. The heuristic uses genes (items) that represent a certain variable and links them to a chromosome (scale of items). To allow variability, a predefined number of chromosomes are then randomly generated from the original item pool, which represents the 1st generation of items (usually 100–200 individuals; Yarkoni, 2010). The algorithm now pursues the goal of maximizing the psychometric quality of the questionnaire by evaluating the chromosomes against a “fitness” function. Based on the defined fitness function, each generation’s fittest chromosomes (item sets) are extracted and used as a basis for the next generation (enabling the selection process of the fittest items). To enable the process of variation and establish genetic diversity and mutation, the spontaneous exchange of items within a scale or between two scales is permitted. With a predefined number of iterations, this procedure identifies the fittest chromosome (item combination) with the highest quality (Schroeders et al., 2016).

Thus, we reduced the initial questionnaire to an economic version (11 factors à, three items).^6^ The collected dataset was randomly divided into a training and a test dataset using holdout-validation (*n_training_* = 450; *n_test_* = 403). In the training dataset, the item combinations were examined against a fitness/quality function based on the Chi-Square test statistic, Root-Mean-Square Error of Approximation (RMSEA), Standardized Root-Mean-Square Residual (SRMR), Comparative Fit Index (CFI), and the reliability of the subscales. Additionally, the function includes the assumed latent variable correlations according to the circular structure of the theory of basic human values. The final selected items will be analyzed for their factorial validity in cross-validation (R-package: stuart, function: cross validate) to the test data set. To check the fit of the factorial measurement model to training and test data, a CFA will be conducted in R (package: lavaan; estimator: MLR). Furthermore, measurement invariance will be evaluated between gender and age groups (Meredith, 1993). Using the cross validate function of the stuart-package, the tests for measurement invariance are not conducted sequentially as often applied (Cheung and Rensvold, 2002). In the approach given by stuart (Schultze, 2017), a measurement model with strict measurement invariance is assumed *a priori*. Therefore, any discrepancy between the scale and the assumed measurement model would manifest in the overall model fit. Due to non-normal data distributions in various scales, a robust estimator was used (skew and kurtosis in Mardia’s Test of multivariate normality, e.g., Authority: skew = −0.15, kurtosis = −0.57; Social Justice: skew = −0.82, kurtosis = 0.99). The fit indices are reported according to Kline (2016). Cutoffs are derived from Gäde et al. (2020, p. 649) for good and acceptable model fit with heterogeneous items.^7^ The circular theoretical ordering of work values (Schwartz et al., 2012; Albrecht et al., 2020) is to be identified per non-metric MDS (R-package: MASS). The aim is to display the correlation-based distances between the work values from a higher dimensional ordering on a two-dimensional space using an iterative estimation to reduce the stress value (Kruskal, 1964; Hout et al., 2013).

To analyze convergent and discriminant validity, latent and manifest correlations between work values and motives, neuroticism, and environmental awareness are considered. As values tend to be somewhat important in general (Sagiv and Schwartz, 2022) and individuals may differ in their response styles (Rudnev, 2021), a common variance factor is important to consider when analyzing personal values. Manifest correlations among work values and basic values will be assessed using ipsative, intraindividual mean-centered scores. For the theoretical assumptions of the theory of basic human values and its circularity, the application of this approach to control for common factor variance can be beneficial. Ipsatization converges ratings to preferences which is more aligned with the theoretical definition of values. Scores based on ipsatized data may be more resistant to common factor bias of response styles and social desirability (Rudnev, 2021). Problems with ipsatization can occur when estimating internal consistency, test–retest reliability or using multivariate techniques due to perfect collinearity. However, we only use ipsatization to assess convergent validity in bivariate correlations to test theoretical assumptions in the nomological net of work values and basic values. Regarding latent variables, bifactor models are potential procedures to control for individual response styles and common variance (Rudnev, 2021). However, only limited empirical evidence for their application in the context of human values is present (e.g., Lilleoja et al., 2016), and their usage must still be thoroughly evaluated (Mansolf and Reise, 2017). Nevertheless, for transparency issues, we report a bifactor model for our final work value scale based on the whole data set to estimate the variance of a potential common variance factor. Therefore, we included an extra method, g-factor, which has equal loadings for each item and is unrelated to the other factors (Schwartz et al., 2012). In this model, we fixed the factor loadings of the first indicator to 1, based on previous bifactor models in value research (Lilleoja et al., 2016).

For the evaluation of our hypothesis, differences in the magnitude of Pearson correlations will be evaluated. The confidence interval (CI) of the difference between Pearson correlations (based on Fisher’s *r*-to-*z* transformation) will be calculated to compare the associations (Zou, 2007; R-package: cocor). An upper level of the CI below zero indicates that the two correlations are not equal (e.g., Authority is lower correlated with social work motives than Social Justice). Due to power considerations, we assess discriminant validity with the confidence interval of the latent correlations in CFAs [CI_CFA_(sys)]. In the analysis, the variances of the latent variables will be fixed to 1. We will inspect the upper/lower limits of the 95% CI of the estimated factor correlations according to Rönkkö and Cho (2022).

The incremental validity of the individual scales is assessed via hierarchical regressions and the additional explained variance (Δ*R*^2^*_adjusted_*). The *R*^2^*_adjusted_* of the baseline model (consisting of one basic value dimension) is considered in comparison to the regression model with the corresponding work values (*cf.* Table 1). The prerequisites are examined according to Bühner and Ziegler (2009). Due to violations of normality assumptions assessed with Shapiro–Wilk test and heteroscedasticity assessed via Breusch–Pagan test, the regressions were performed via Bootstrapping (5,000 iterations). Based on the criticism of using conventional significance levels (Kline, 2019) for evaluating *p*-values, we calculate an Alpha that minimizes Type I and II errors (Mudge et al., 2012), considering the smallest effect size of interest (*r* = 0.30; *ƒ*^2^ = 0.10; *α*_correlation_ = 0.001; *α*_regression_ = 0.001). This makes interpretation less arbitrary and more adjusted to context and data (R-package: JustifyAlpha; Maier and Lakens, 2022).

## 4 Results

Descriptive statistics of assessed scales are displayed in Tables 4 and 5. We conducted CFAs (estimator: MLR) for the work motive questionnaire (χ^2^ = 1333.86, *df* = 134, *p* < 0.001, χ^2^/*df* = 9.95, CFI = 0.771, RMSEA = 0.102 [0.098, 0.107], and SRMR = 0.073), and the basic value questionnaire (χ^2^ = 459.86, *df* = 113, *p* < 0.001, χ^2^/*df* = 4.07, CFI = 0.908, RMSEA = 0.06 [0.055, 0.065], and SRMR = 0.07). Neuroticism, environmental awareness, and value congruence were collectively analyzed with constrained covariances (χ^2^ = 176.62, *df* = 44, *p* < 0.001, χ^2^/*df* = 4.01, CFI = 0.964, RMSEA = 0.059 [0.051, 0.068], and SRMR = 0.083). The work motive questionnaire especially shows weak factorial validity.

**Table 4:** Descriptive statistics and ipsatized Pearson correlations of work values (*n* = 853).

	*M* (SD)	1		2		3		4		5		6		7		8		9		10		11
1. Authority	4.10 (1.41)	1																				
2. Ambition	5.04 (1.12)	0.20***	[0.13, 0.26]	1																		
3. Enjoyment	5.87 (0.79)	−0.41***	[−0.47, −0.35]	−0.17***	[−0.24, −0.11]	1																
4. Variety	5.51 (0.98)	−0.05	[−0.11, 0.02]	0.03	[−0.03, 0.10]	0.10**	[0.03, 0.17]	1														
5. Autonomy	5.63 (0.86)	−0.06*	[−0.13, 0.01]	−0.08**	[−0.14, −0.01]	0.26***	[0.20, 0.32]	0.25***	[0.19, 0.31]	1												
6. Social Justice	5.38 (1.09)	−0.25***	[−0.31, −0.18]	−0.28***	[−0.34, −0.22]	−0.11***	[−0.18, −0.05]	−0.22***	[−0.28, −0.16]	−0.23***	[−0.29, −0.16]	1										
7. Environmental Sustainability	4.83 (1.48)	−0.23***	[−0.29, −0.17]	−0.29***	[−0.35, −0.23]	−0.19***	[−0.25, −0.12]	−0.26***	[−0.32, −0.19]	−0.34***	[−0.40, −0.28]	0.28***	[0.22, 0.34]	1								
8. Helping and Supporting	5.32 (1.10)	−0.29***	[−0.35, −0.23]	−0.26***	[−0.32, −0.20]	0.06*	[−0.01, 0.13]	−0.11***	[−0.18, −0.05]	−0.23***	[−0.29, −0.17]	0.23***	[0.17, 0.29]	0.10**	[0.03, 0.17]	1						
9. Rule Respecting	5.42 (0.96)	−0.27***	[−0.34, −0.21]	−0.04	[−0.11, 0.03]	0.12***	[0.05, 0.18]	−0.08**	[−0.15, −0.01]	−0.05	[−0.12, 0.02]	−0.15***	[−0.21, −0.08]	−0.27***	[−0.33, −0.21]	−0.15***	[−0.22, −0.09]	1				
10. Traditional Values	4.95 (1.07)	−0.02	[−0.09, 0.04]	−0.12***	[−0.18, −0.05]	−0.11***	[−0.18, −0.05]	−0.30***	[−0.36, −0.24]	−0.10**	[−0.17, −0.04]	−0.16***	[−0.22, −0.09]	−0.14***	[−0.20, −0.07]	−0.14***	[−0.20, −0.07]	0.11**	[0.04, 0.17]	1		
11. Safety	5.24 (1.02)	−0.19***	[−0.25, −0.12]	−0.04	[−0.11, 0.02]	−0.07*	[−0.13, 0.00]	−0.22***	[−0.28, −0.15]	−0.20***	[−0.26, −0.13]	−0.06*	[−0.13, 0.01]	−0.07*	[−0.14, −0.00]	−0.08*	[−0.14, −0.01]	0.15***	[0.08, 0.21]	0.03	[−0.03, 0.10]	1

[95% CI], * < 0.05; ** < 0.01; *** < 0.001; α-level to assess significance: <0.001.

**Table 5:** Pearson correlations of work values and variables from the nomological network (*n* = 853).

	*M* (SD)	Authority	Ambition	Enjoyment	Variety	Autonomy	Social justice	Environmental sustainability	Helping and Supporting	Rule respecting	Traditional values	Safety	Conservation	Openness to Change	Self-Enhancement		Self-Transcendence		Individualistic work motives		Social work motive		Environmental awareness		Neuroticism		Value congruence
Conservation	4.47 (0.91)	−0.09** [−0.15, −0.02]	0.02 [−0.05, 0.09]	−0.04 [−0.11, 0.02]	−0.07* [−0.14, −0.00]	−0.04 [−0.11, 0.03]	−0.17*** [−0.23, −0.10]	−0.11** [−0.18, −0.04]	−0.08** [−0.15, −0.02]	0.26*** [0.20, 0.32]	0.28*** [0.21, 0.34]	0.16*** [0.09, 0.22]	1														
Opennes to change	4.87 (0.78)	−0.27*** [−0.33, −0.21]	−0.07* [−0.13, −0.00]	0.23*** [0.17, 0.29]	0.27*** [0.21, 0.33]	0.25*** [0.18, 0.31]	0 [−0.07, 0.06]	−0.04 [−0.11, 0.03]	−0.04 [−0.11, 0.02]	0.04 [−0.03, 0.11]	−0.12*** [−0.19, −0.05]	−0.03 [−0.09, 0.04]	−0.33*** [−0.39, −0.27]	1													
Self-enhancement	3.58 (0.98)	0.57*** [0.52, 0.61]	0.36*** [0.30, 0.42]	−0.21*** [−0.27, −0.14]	−0.03 [−0.09, 0.04]	−0.02 [−0.09, 0.04]	−0.22*** [−0.28, −0.16]	−0.22*** [−0.28, −0.15]	−0.16*** [−0.23, −0.10]	−0.18*** [−0.25, −0.12]	−0.04 [−0.10, 0.03]	−0.16*** [−0.22, −0.09]	−0.18*** [−0.24, −0.11]	−0.51*** [−0.55, −0.45]	1												
Self-transcendence	4.81 (0.83)	−0.42*** [−0.47, −0.36]	−0.41*** [−0.46, −0.35]	0.10** [0.04, 0.17]	−0.14*** [−0.20, −0.07]	−0.14*** [−0.21, −0.08]	0.40*** [0.35, 0.46]	0.39*** [0.33, 0.44]	0.30*** [0.24, 0.36]	0 [−0.07, 0.06]	−0.07* [−0.13, 0.00]	0.10** [0.03, 0.16]	−0.27*** [−0.33, −0.21]	0.06* [−0.01, 0.13]	−0.68*** [−0.72, −0.65]		1										
Individualistic work motives	3.76 (0.60)	0.50*** [0.45, 0.55]	0.67*** [0.63, 0.71]	0.55*** [0.50, 0.59]	0.68*** [0.65, 0.72]	0.61*** [0.57, 0.65]	0.56*** [0.52, 0.61]	0.39*** [0.34, 0.45]	0.54*** [0.49, 0.58]	0.50*** [0.45, 0.55]	0.52*** [0.47, 0.57]	0.56*** [0.51, 0.60]	0.25*** [0.19, 0.31]	0.41*** [0.35, 0.46]	0.34*** [0.28, 0.40]		0.35*** [0.28, 0.40]		1								
Social work motives	3.16 (0.74)	0.48*** [0.43, 0.53]	0.50*** [0.44, 0.55]	0.40*** [0.34, 0.45]	0.44*** [0.38, 0.49]	0.24*** [0.18, 0.30]	0.55*** [0.51, 0.60]	0.46*** [0.41, 0.51]	0.63*** [0.58, 0.66]	0.38*** [0.32, 0.44]	0.52*** [0.47, 0.57]	0.54*** [0.49, 0.58]	0.20*** [0.14, 0.26]	0.13*** [0.06, 0.19]	0.27*** [0.21, 0.33]		0.24*** [0.18, 0.31]		0.61*** [0.56, 0.65]		1						
Evironmental awareness	4.58 (0.96)	−0.06* [−0.13, 0.01]	0.08** [0.02, 0.15]	0.31*** [0.25, 0.37]	0.17*** [0.10, 0.23]	0.12*** [0.06, 0.19]	0.36*** [0.30, 0.42]	0.52*** [0.47, 0.56]	0.27*** [0.21, 0.33]	0.25*** [0.19, 0.31]	0.17*** [0.10, 0.23]	0.25*** [0.18, 0.31]	0.09** [0.03, 0.16]	0.25*** [0.19, 0.31]	−0.15*** [−0.22, −0.08]		0.45*** [0.40, 0.50]		0.18*** [0.11, 0.24]		0.15*** [0.08, 0.21]		1				
Neuroticism	3.51 (1.41)	−0.21*** [−0.27, −0.14]	−0.11** [−0.18, −0.04]	0 [−0.06, 0.07]	−0.16*** [−0.22, −0.09]	−0.16*** [−0.23, −0.09]	−0.05 [−0.12, 0.01]	−0.02 [−0.09, 0.05]	−0.02 [−0.09, 0.05]	−0.09** [−0.15, −0.02]	−0.14** [−0.21, −0.07]	−0.08* [−0.14, −0.01]	−0.13** [−0.19, −0.06]	−0.16** [−0.23, −0.09]	−0.06* [−0.13, 0.00]		0.04 [−0.03, 0.11]		−0.16*** [−0.23, −0.10]		−0.10** [−0.17, −0.03]		0.09** [0.02, 0.16]		1		
Value congruence	4.84 (1.20)	0.29*** [0.23, 0.35]	0.39*** [0.34, 0.45]	0.32*** [0.25, 0.37]	0.31*** [0.25, 0.37]	0.23*** [0.17, 0.29]	0.38*** [0.32, 0.43]	0.29*** [0.23, 0.35]	0.40*** [0.34, 0.45]	0.43*** [0.37, 0.48]	0.45*** [0.39, 0.50]	0.38*** [0.32, 0.44]	0.26*** [0.20, 0.32]	0.18*** [0.11, 0.24]	0.19*** [0.13, 0.26]		0.27*** [0.21, 0.33]		0.42*** [0.36, 0.47]		0.39*** [0.33, 0.45]		0.10** [0.03, 0.16]		−0.21*** [−0.27, −0.14]		1

[95% CI], * < 0.05; ** < 0.01; *** < 0.001; α-level to assess significance: <0.001; correlations between work values, basic values, and among each other are based on ipsative scores.

### 4.1 Model fit and latent structure of the CWVS

The final set of selected items (see Table 6) shows a good fit for the training data. Cross-validation of the test data supports the assumption of strict measurement invariance between the two samples (see Table 7). Additionally, the analysis of measurement invariance between age and gender groups is in line with the conjecture of strict measurement invariance. Hence, the data support our first hypothesis. The bifactor model shows a 10% variance in the common variance factor with a good fit of the model to the data (see Table 7).

**Table 6:** Final set of selected items in the CWVS in German and English wording, including standardized factor loadings, standard errors, and reliability estimates.

Value construct	German wording	English wording	Factor loadings	Standard errors	Bifactor model		
			Training/Test		Factor loadings	Standard errors	Loading on g-factor
Authority/Autorität α = 0.80; ω = 0.81	1. Andere Menschen führen können	Be able to lead other people	0.82/0.81	0.07/0.07	0.77		0.32
	2. Bestimmen, wie Geld ausgegeben wird	Determine how money is spent ^a^	0.71/0.70	0.07/0.08	0.66	0.05	0.31
	3. Entscheidungen darüber treffen können, wer welche Aufgaben übernimmt	Make decisions about who does what ^a^	0.75/0.76	0.07/0.08	0.69	0.05	0.34
Ambition/Ehrgeiz α = 0.78; ω = 0.79	4. In der Organisation als erfolgreich angesehen werden	Be seen as successful in the organization	0.66/0.69	0.07/0.07	0.56		0.40
	5. Ehrgeizig sein	Be ambitious ^a^	0.75/0.79	0.06/0.07	0.71	0.13	0.37
	6. Leistung zeigen können	Be able to show performance	0.78/0.79	0.06/0.06	0.63	0.10	0.48
Enjoyment/ Vergnügen α = 0.66; ω = 0.68	7. Freude empfinden	Have fun ^a^	0.62/0.66	0.04/0.07	0.38		0.55
	8. Ausgleich zwischen beruflichen und erholsamen Tätigkeiten	Balance professional and recreational activities	0.49/0.49	0.06/0.08	0.10	0.25	0.54
	9. Dinge tun, die mir ein gutes Gefühl geben	Do things which make me feel good ^a^	0.74/0.81	0.04/0.06	0.61	0.37	0.54
Variety/ Abwechslung α = 0.85; ω = 0.85	10. Abwechslungsreiche Aufgaben haben	Do varied work ^a^	0.82/0.79	0.05/0.06	0.62		0.51
	11. Eine Vielfalt an Aufgaben bearbeiten	Experience a wide variety of tasks ^a^	0.78/0.78	0.05/0.06	0.63	0.08	0.49
	12. Abwechslungsreiche Herausforderungen erleben	Experience a variety of challenges ^a^	0.83/0.81	0.05/0.06	0.70	0.09	0.47
Autonomy/ Autonomie α = 0.75; ω = 0.76	13. Entscheiden, wie ich meine Aufgaben erledige	Be able to direct my own work ^a^	0.76/0.63	0.04/0.05	0.40		0.56
	14. Meine eigenen Prioritäten bei der Arbeit setzen	Decide my own priorities at work ^a^	0.67/0.74	0.05/0.06	0.46	0.20	0.50
	15. Selbstständig und eigenverantwortlich handeln können	Be able to act independently and on my own responsibility	0.76/0.72	0.04/0.05	0.52	0.12	0.55
Social Justice/ Soziale Gerechtigkeit α = 0.82; ω = 0.83	16. Mich für einen respektvollen Umgang in der Organisation einsetzen	To promote respectful behavior in the organization	0.77/0.75	0.06/0.07	0.60		0.47
	17. Zur Fairness in der Organisation beitragen	Contribute to fairness in the organization	0.83/0.80	0.06/0.07	0.68	0.08	0.45
	18. Mich für Chancengleichheit der Kolleg:innen in meinem Arbeitsumfeld einsetzen	To work for equal opportunities for colleagues in my work environment	0.76/0.78	0.07/0.07	0.70	0.14	0.39
Environmental Sustainability/ Ökologische Nachhaltigkeit α = 0.92; ω = 0.93	19. Die Umwelt schützen	Protect the environment ^a^	0.93/0.89	0.06/0.07	0.85		0.35
	20. In einer Organisation arbeiten, die den Umweltschutz unterstützt	Work in an organization that supports environmental protection	0.88/0.85	0.06/0.07	0.80	0.03	0.34
	21. Umweltbewusst handeln	Act in an environmentally conscious way	0.92/0.92	0.06/0.06	0.84	0.03	0.36
Helping and Supporting/ Helfen und Unterstützen α = 0.85; ω = 0.85	22. Menschen helfen, mit denen ich in Kontakt komme	Help the people I come in contact ^a^	0.84/0.87	0.05/0.05	0.71		0.47
	23. Anderen Menschen durch meine Arbeit helfen	Do work which helps other people ^a^	0.77/0.80	0.06/0.06	0.67	0.06	0.43
	24. Das Leben der Menschen, denen ich bei der Arbeit begegne, verbessern	Improve the lives of people I encounter at work ^a^	0.78/0.78	0.06/0.06	0.67	0.07	0.42
Rule Respecting/ Regeln Respektieren α = 0.79; ω = 0.79	25. In einem Team arbeiten, in dem wir alle die Richtlinien der Organisation unterstützen	Work in a group where we all support the organization’s policies ^a^	0.78/0.79	0.05/0.07	0.64		0.48
	26. An einem Arbeitsplatz arbeiten, an dem Regeln eingehalten werden	Work in a workplace where rules are respected	0.76/0.71	0.05/0.08	0.53	0.11	0.51
	27. Mit Kolleg:innen zusammenarbeiten, die die Regeln auch dann einhalten, wenn niemand sie beobachtet	Work with colleagues who respect rules even when no one else sees them ^a^	0.66/0.76	0.05/0.07	0.50	0.11	0.48
Traditional Values/ Traditionelle Werte α = 0.61; ω = 0.61	28. Im Einklang mit den Überzeugungen meiner Familie zu arbeiten	Be able to work according to the values of my family ^a^	0.55/0.45	0.07/0.09	0.33		0.39
	29. Eine Arbeit verrichten, die mit meinen kulturellen Werten übereinstimmt	Do work that is consistent with my cultural values	0.59/0.54	0.06/0.08	0.41	0.20	0.40
	30. Die Traditionen meiner Organisation fortführen	To carry on the traditions of my organization	0.70/0.65	0.07/0.07	0.61	0.30	0.38
Safety/Sicherheit α = 0.71; ω = 0.71	31. Zur Sicherheit meiner Kolleg:innen beitragen	Contribute to the safety of my colleagues ^a^	0.75/0.77	0.06/0.06	0.67		0.42
	32. Die Sicherheit des Arbeitsplatzes maximieren	Maximize job security	0.62/0.68	0.06/0.07	0.45	0.08	0.45
	33. Unterstützende Sozial- & Zusatzleistungen bereitgestellt durch die Organisation	Supportive social and fringe benefits provided by the organization	0.54/0.61	0.07/0.07	0.37	0.09	0.44

Initial question: “Regardless of your current job, how important are the following aspects to you personally at work?”; English wording derived through back-translation approach or derived from Albrecht et al. (2020)^a^.

**Table 7:** Results of model comparisons in cross-validation and bifactor model.

	χ2(df)	χ2/df	CFI	RMSEA [90%-CI]	SRMR
Datasets					
Training	591.85(440)	1.35	0.973	0.029 [0.023, 0.033]	0.039
Test	681.32(440)	1.55	0.954	0.037 [0.032, 0.042]	0.045
Configural	1705.36(880)	1.94	0.944	0.047	0.045
Metric	1726.71(902)	1.91	0.944	0.046	0.045
Scalar	1777.08(935)	1.90	0.943	0.046	0.046
Strict	1862.83(968)	1.92	0.939	0.047	0.046
Gender					
Women	682.29(440)	1.55	0.955	0.036 [0.032, 0.041]	0.043
Men	753.31(440)	1.71	0.948	0.040 [0.036; 0.045]	0.043
Configural	1793.04(880)	2.04	0.938	0.049	0.043
Metric	1834.39(902)	2.03	0.937	0.049	0.046
Scalar	1920.85(935)	2.05	0.933	0.050	0.049
Strict	2011.54(968)	2.08	0.929	0.050	0.050
Age					
18–43	633.51(440)	1.44	0.957	0.035 [0.029, 0.040]	0.048
44–69	761.88(440)	1.73	0.954	0.039 [0.035, 0.043]	0.040
Configural	1747.70(880)	1.99	0.941	0.048	0.043
Metric	1786.37(902)	1.98	0.940	0.048	0.045
Scalar	1962.47(935)	2.10	0.931	0.051	0.049
Strict	2081.32(968)	2.15	0.925	0.052	0.049
Bifactor model	781.2(439)	1.78	0.969	0.034 [0.030, 0.038]	0.052

**Figure 1:**
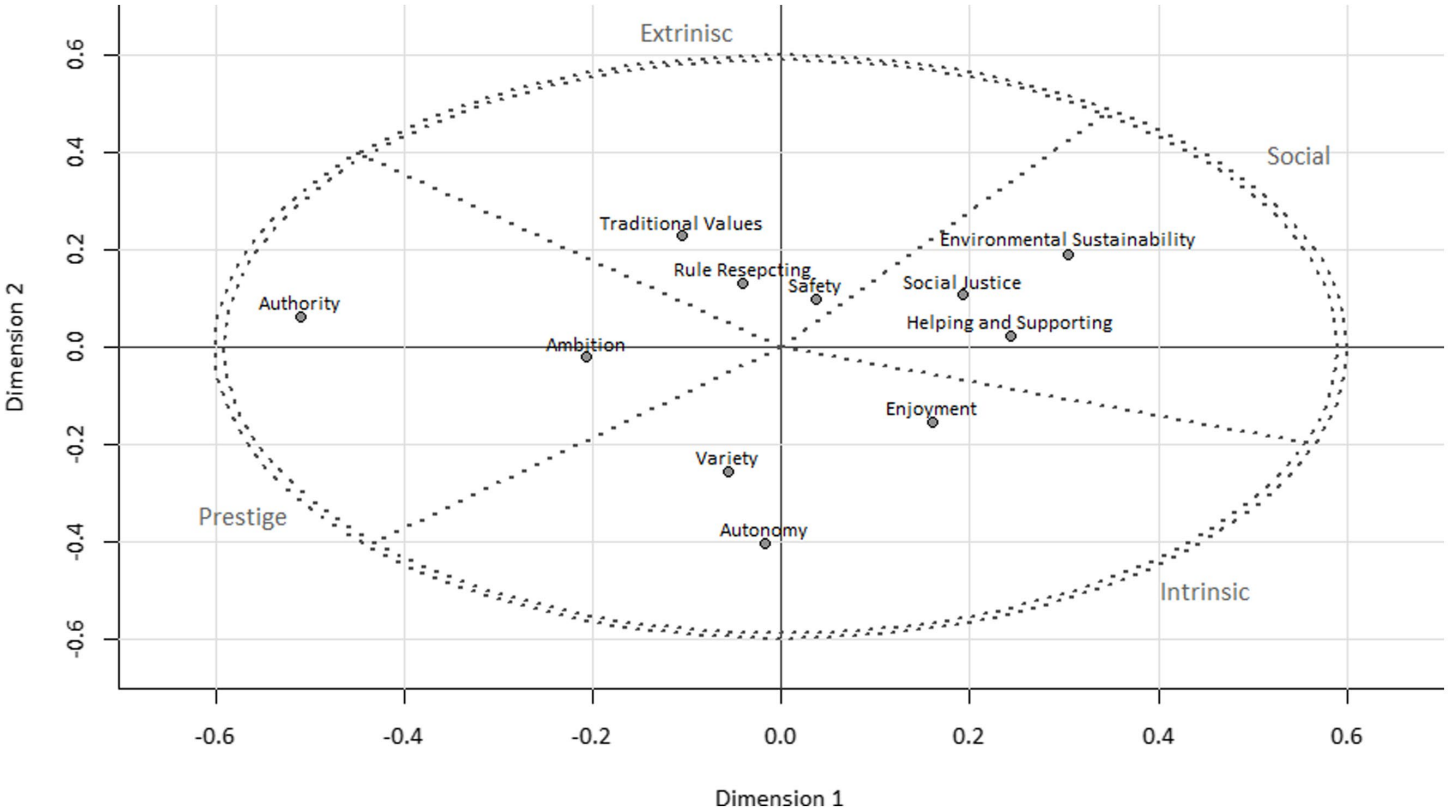
MDS to assess the dimensionality and theoretical circularity of the work values.

The stress index of the final MDS solution (stress = 0.13; see Figure 1) is less than the recommended criterion of 0.15 (Dugard et al., 2010), suggesting goodness of fit. Each quadrant of the ellipses includes one work value dimension supporting the circularity and the relationships of opposing value dimensions. Thus, Hypothesis 2 is supported.

### 4.2 Analysis of convergent and discriminant validity

Latent and Pearson correlation statistics are displayed in Tables 4, 5 and 8. Convergent validity is assessed with correlations from Table 5. Compared to the two Prestige work values Ambition and Authority, Social Justice (*r_Diff-Authority_* = −0.07 [−0.14, −0.01], *r_Diff-Ambition_* = −0.06 [−0.11, −0.002]) and Helping and Supporting (*r_Diff-Authority_* = −0.14 [−0.21, −0.08], *r_Diff-Ambition_* = −0.13 [−0.18, −0.07]) exhibit higher Pearson correlations with social work motives, while Environmental Sustainability (*r_Diff-Authority_* = 0.02 [−0.05, 0.09], *r_Diff-Ambition_* = 0.04 [−0.03, 0.10]) shows similar correlations. Correspondingly, Autonomy (*r_Diff-Rule Respecting_* = −0.11 [−0.17, −0.05], *r_Diff-Traditional Values_* = −0.09 [−0.15, −0.03], *r_Diff-Safety_* = −0.06 [−0.11, −0.002]), and Variety (*r_Diff-Rule Respecting_* = −0.18 [−0.23, −0.13], *r_Diff-Traditional Values_* = −0.16 [−0.22, −0.11], *r_Diff-Safety_* = −0.13 [−0.18, −0.08]) demonstrate higher Pearson correlations with individualistic work motives, whereas Enjoyment (*r_Diff-Rule Respecting_* = −0.04 [−0.10, 0.01], *r_Diff-Traditional Values_* = −0.02 [−0.08, 0.03], *r_Diff-Safety_* = 0.01 [−0.04, 0.06]) as part of the Intrinsic work values and Rule Respecting, Traditional Values, and Safety exhibit lower correlations. The *p*-value of the latent and Pearson associations is lower than the computed optimal Alpha of 0.001. Conclusively, Hypotheses 3a and 3b are partially supported by our data as the work values Enjoyment and Environmental Sustainability are not strongly related to the corresponding work motives.

**Table 8:** Latent correlations of work values and variables for assessing convergent and discriminant validity.

	Conservation		Openness to change		Self-enhancement		Self-transcendence		Individualistic work motives		Social work motive		Environmental awareness		Neuroticism	
Authority	0.20***	[0.09, 0.31]	0.11*	[0.01, 0.20]	0.75***	[0.68, 0.82]	0.07	[−0.03, 0.17]	0.63***	[0.57, 0.69]	0.54***	[0.46, 0.61]	−0.02	[−0.11, 0.07]	−0.23***	[−0.32, −0.14]
Ambition	0.50***	[0.40, 0.60]	0.43***	[0.34, 0.52]	0.64***	[0.56, 0.72]	0.35***	[0.26, 0.45]	0.81***	[0.77, 0.86]	0.58***	[0.51, 0.65]	0.13**	[0.05, 0.22]	−0.13**	[−0.21, −0.04]
Enjoyment	0.43***	[0.33, 0.52]	0.50***	[0.41, 0.59]	0.21***	[0.12, 0.30]	0.60***	[0.52, 0.68]	0.66***	[0.59, 0.72]	0.58***	[0.51, 0.65]	0.41***	[0.33, 0.49]	0	[−0.08, 0.09]
Variety	0.37***	[0.27, 0.47]	0.52***	[0.42, 0.61]	0.27***	[0.18, 0.35]	0.45***	[0.36, 0.54]	0.79***	[0.75, 0.83]	0.53***	[0.46, 0.59]	0.23***	[0.14, 0.31]	−0.18***	[−0.26, −0.10]
Autonomy	0.33***	[0.24, 0.42]	0.48***	[0.40, 0.57]	0.34***	[0.25, 0.42]	0.35***	[0.26, 0.44]	0.74***	[0.68, 0.79]	0.30***	[0.22, 0.38]	0.18***	[0.09, 0.27]	−0.19***	[−0.27, −0.10]
Social Justice	0.26***	[0.16, 0.35]	0.30***	[0.22, 0.39]	0.13**	[0.04, 0.22]	0.58***	[0.49, 0.68]	0.63***	[0.57, 0.69]	0.75***	[0.70, 0.80]	0.45***	[0.37, 0.53]	−0.06	[−0.14, 0.03]
Environmental Sustainability	0.25***	[0.16, 0.35]	0.26***	[0.18, 0.34]	0.14**	[0.04, 0.23]	0.52***	[0.40, 0.63]	0.42***	[0.35, 0.49]	0.57***	[0.50, 0.64]	0.61***	[0.54, 0.67]	−0.02	[−0.10, 0.07]
Helping and Supporting	0.37***	[0.28, 0.46]	0.40***	[0.31, 0.49]	0.06	[−0.04, 0.15]	0.58***	[0.49, 0.66]	0.59***	[0.53, 0.66]	0.85***	[0.82, 0.89]	0.34***	[0.25, 0.42]	−0.02	[−0.10, 0.07]
Rule Respecting	0.58***	[0.49, 0.67]	0.43***	[0.35, 0.51]	0.20***	[0.11, 0.29]	0.51***	[0.43, 0.60]	0.56***	[0.48, 0.64]	0.50***	[0.42, 0.58]	0.31***	[0.22, 0.39]	−0.11*	[−0.19, −0.02]
Traditional Values	0.62***	[0.51, 0.74]	0.17***	[0.09, 0.26]	0.38***	[0.29, 0.47]	0.25***	[0.16, 0.35]	0.69***	[0.62, 0.76]	0.74***	[0.67, 0.81]	0.24***	[0.14, 0.34]	−0.18**	[−0.29, −0.08]
Safety	0.56***	[−47, 0.66]	0.42***	[0.33, 0.51]	0.25***	[0.16, 0.34]	0.60***	[0.50, 0.69]	0.67***	[0.60, 0.73]	0.73***	[0.67, 0.79]	0.36***	[0.27, 0.44]	−0.11*	[−0.21, −0.02]

[95% CI], * < 0.05; ** < 0.01; *** < 0.001; latent variable correlations in upper half calculated through CFAs.

For Hypothesis 4, we inspected the correlation of the newly set up work value Environmental Sustainability with the construct of environmental awareness. Latent and Pearson correlations (*r_latent_* = 0.61, *r_Pearson_* = 0.52) support our assumption.

Discriminant validity is evaluated by analyzing the relations of the work values measured with the CWVS and the Big Five-dimension neuroticism (H5). The CI_CFA_(sys) of the latent correlations are displayed in Table 8. Work values of Authority (*r_latent_* = −0.23 [−0.32, −0.14]), Variety (*r_latent_* = −0.18 [−0.26, −0.10]) and Autonomy (*r_latent_* = −0.19 [−0.27, −0.10]) demonstrate significant negative latent correlations to neuroticism (*p* < 0.001). Nonetheless, the limits of the intervals are below the recommended Cutoff of 0.80. Therefore, empirical distinction according to Hypothesis 5 is supported.

### 4.3 Congruent incremental validity

For the sixth hypothesis, we conducted hierarchical linear regressions. The aim is to assess the additional explained variance of the work values compared to one basic value dimension in the criterion of value congruence. The results are displayed in Table 9. The inclusions of Social (Δ*R*^2^_adjusted_ = 0.10), Prestige (Δ*R*^2^_adjusted_ = 0.13), Intrinsic (Δ*R*^2^_adjusted_ = 0.10), and Extrinsic (Δ*R*^2^_adjusted_ = 0.18) work values show significant increases in the explained variance, supporting Hypothesis 6.

**Table 9:** Results of hierarchical linear regression analysis for the prediction of the value congruence and change in the adjusted *R*^2^.

		*Model 1*		*Model 2*	
Model information	Predictor	*β* [95% CI]	*SE*	*β* [95% CI]	*SE*
*ΔR^2^_adjusted_ = 0.10* *F* for *R*^2^_change_ = 37.03***	Self-Transcendence	0.41*** [0.30, 0.52]	0.06	0.11* [−0.002, 0.23]	0.06
	Gender	−0.14 [−0.29, 0.02]	0.08	−0.13 [−0.28, 0.02]	0.08
	Age	−0.004 [−0.01, 0.003]	0.004	−0.004 [−0.01, 0.003]	0.003
	Tenure	0.002 [−0.01, 0.01]	0.004	0.004 [−0.005, 0.01]	0.004
	Social Justice			0.17** [0.05, 0.30]	0.06
	Environmental Sustainability			0.01 [−0.07, 0.09]	0.04
	Helping and Supporting			0.28*** [0.17, 0.38]	0.05
*ΔR^2^_adjusted_* = 0.13 *F* for *R*^2^_change_ = 67.15***	Self-Enhancement	0.24*** [0.14, 0.34]	0.05	−0.08 [−0.19, 0.03]	0.06
	Gender	0.001 [−0.16, 0.17]	0.08	0.04 [−0.11, 0.19]	0.08
	Age	0.001 [−0.01, 0.01]	0.003	0.003 [−0.003, 0.01]	0.003
	Tenure	0.00 [−0.01, 0.01]	0.004	0.00 [−0.01, 0.01]	0.004
	Authority			0.12*** [0.05, 0.20]	0.04
	Ambition			0.38*** [0.29, 0.47]	0.05
*ΔR^2^_adjusted_* = 0.10 *F* for *R*^2^_change_ = 32.65***	Openness to Change	0.29*** [0.17, 0.39]	0.06	0.02 [−0.08, 0.13]	0.05
	Gender	−0.08 [−0.24, 0.09]	0.08	−0.13 [−0.28, 0.02]	0.08
	Age	−0.003 [−0.01, 0.004]	0.004	−0.003 [−0.01, 0.004]	0.004
	Tenure	0.001 [−0.01, 0.01]	0.004	−0.002 [−0.01, 0.01]	0.004
	Variety			0.23*** [0.12, 0.35]	0.06
	Enjoyment			0.32*** [0.19, 0.45]	0.06
	Autonomy			0.02 [−0.11, 0.15]	0.07
*ΔR^2^_adjusted_* = 0.18 *F* for *R*^2^_change_ = 69.32***	Conservation	0.36*** [0.26, 0.46]	0.05	0.04 [−0.05, 0.14]	0.05
	Gender	−0.04 [−0.20, 0.11]	0.08	−0.03 [−0.17, 0.11]	0.07
	Age	−0.01 [−0.01, 0.002]	0.004	−0.01 [−0.01, 0.001]	0.003
	Tenure	−0.001 [−0.01, 0.01]	0.004	−0.00 [−0.01, 0.01]	0.004
	Rule Respecting			0.28*** [0.14, 0.40]	0.07
	Traditional Values			0.31*** [0.21, 0.41]	0.05
	Safety			0.08 [−0.03, 0.20]	0.06

α-level for assessing significance in Δ*R*^2^_adjusted_: α = 0.001; ***p < 0.001, **p < 0.01, *p < 0.05; gender was dummy-coded for women = 1; dependent variable: Value congruence (PO-Fit).

### 4.4 Exploratory analysis

Due to the weak factorial validity of the IEA-K (Kanning, 2016), we decided to provide additional evidence for convergent validity. We analyzed latent and ipsative Pearson correlations of the CWVS with the basic value dimensions from the Higher-Order Value Scale-17 (Lechner et al., 2022).

As Table 5 illustrates, the latent and ipsative Pearson correlations in the circumplex of basic and work value dimensions support the theoretical assumptions. Prestige work values show positive Pearson/latent correlations with the basic value dimension of Self-Enhancement and negative Pearson and lower latent correlations with Self-Transcendence. The opposite accounts for Social work values. Intrinsic work values also correlate more positively with Openness to Change and negatively, respectively, lower with Conservation. Correspondingly, the opposite accounts for Extrinsic work values. As a result, convergent validity is supported by the additional analysis.

## 5 Discussion

The goal of this study was to develop a German-language work value questionnaire with accurate content, factorial and nomological validity that can be utilized in organizational and work-related settings. Based on the theory of basic human values (Schwartz, 1992), a questionnaire developed by Albrecht et al. (2020) was used as a starting point to capture the contextualized 11 work values representing the theoretical circularity of value constructs. The questionnaire development process was based on guidelines for cultural adaption and item generation (MacKenzie et al., 2011; International Test Commission, 2017; Boateng et al., 2018). A cross-sectional panel survey led to the inclusion of *n* = 853 cases. The questionnaire was developed using a genetic algorithm. Convergent, discriminant and congruent incremental validity were assessed, as well as the theoretical representation using MDS.

To our knowledge, this study is the first to support the full application of the theory of basic human values to the German work context. The developed questionnaire provides good psychometric quality with satisfying fit to test and training data and supports the assumption of strict measurement invariance between age groups and gender. The theoretical foundation is supported by MDS, as the work values provide a clear circular structure of conflicting and congruent underlying work-related goals and expectations. The dimension of Safety, which originally was understood as safety climate (Albrecht et al., 2020), is measured with three items, including interpersonal and intrapersonal Safety aspects. This potentially explains why the value can be found in the original Extrinsic domain. The work value of Enjoyment is more distinct from the other Intrinsic values. Implications of this conjuncture are discussed next.

Analysis of relations to work motives and basic values support convergent validity except for Enjoyment and Environmental Sustainability. Enjoyment does not correlate more strongly with individualistic work motives than Extrinsic work values. This may be due to its lower internal consistency as items capture different facets of the underlying work value (having fun at work and balancing work and recreational time). Additionally, as Enjoyment is more distant from Autonomy and Variety in the MDS solution, its theoretical and, therefore, empirical associations in the circumplex might be closer to Social work values. Environmental Sustainability is perhaps not associated with Social work motives beyond Authority and Ambition due to its strict focus on environmental aspects. The value does not capture interpersonal facets and is, therefore, potentially less related to social work motives. However, the MDS solution displays high distances, and ipsative Pearson correlations are significantly negative between Environmental Sustainability and Prestige work values. Nevertheless, it is essential to exercise caution when interpreting the correlations of the work values with work motives, as the short form of the IEA used possesses weak factorial validity in our study. Therefore, the exploratory analysis was conducted, and the basic value dimensions show a clear circular pattern of correlations with the work values. The newly established and more comprehensively assessed work value of Environmental Sustainability correlates moderately to highly positively with the construct of environmental awareness. Hence, convergent validity is backed by our data.

The latent correlations of the work values in the CWVS with the personality construct of neuroticism endorsed our assumption. Based on the 95% CI of latent correlations, the two questionnaires are empirically distinctive. The results are in line with a comprehensive meta-analysis addressing the association between personality traits and personal values (Parks-Leduc et al., 2015).

Finally, we analyzed congruent incremental validity. The additional explained variance of the work values compared to the basic value dimensions in the person–organization fit of value congruence supports our theoretical assumptions. Due to their contextualization, work values tend to provide more content-valid insights, potentially resulting in enhanced criterion validity. This is particularly relevant for work value research as questionnaire development approaches in the past did not study the criterion-related validity of work values compared to basic values (Consiglio et al., 2017; Albrecht et al., 2020; Stiglbauer et al., 2022).

### 5.1 Practical implications

Our study delivers the first evidence that the CWVS is more adequate when predicting work-related outcomes. As the questionnaire’s validity and theoretical assumptions are predominantly supported, we encourage the application of this questionnaire in practical and research settings. The short questionnaire with contextualized items can be used for a thorough assessment of employees’ or applicants’ value structure. The knowledge about what people value at work can provide powerful insights (Arieli et al., 2020; Anglim et al., 2022) and be beneficial for employers and employees (Bojanowska et al., 2022). The face validity of value assessment in work contexts may increase due to the contextualized work values. Hence, the assessment of what employees in an organization expect from their work or to deliver additional material for interventions can be useful applications of work values. For instance, team expectations can be clarified based on the CWVS by enabling a general, work-related framing. Furthermore, work design measures to improve value congruence between tasks and personal values can be adapted more purposefully due to the higher content validity of work values than basic values. The application of work values in career management interventions can provide insights into the personal development of individuals. As some values indirectly tend to affect psychological health, the work-related contextualization of values may be more useful for supporting individuals in their career choices.

### 5.2 Limitations and future research

Although we developed a valid new questionnaire, some limitations need to be acknowledged. The definitive version of the CWVS captures all dimensions quite broadly. Yet, algorithms for item selection only display a heuristic, which does not automatically result in the optimal solution. As we repeated the computation, we found that various scales (Enjoyment, Social Justice, Helping and Supporting, and Safety) were partially unstable. Here, various solutions provided good scale quality. Accordingly, it may be appropriate to discriminate between more narrowly defined work values in correspondence to Schwartz’s refined theory with 19 values. Differentiations in Enjoyment were illustrated previously; Social Justice and Helping and Supporting potentially address colleagues, customers, and the broader society. Additionally, Safety could be further differentiated into interpersonal and intrapersonal safety (complementary to Albrecht et al., 2020), and Traditional Values could be understood as compliance with organizational culture or broader societal culture and norms. Therefore, the use of this questionnaire and the theory of basic human values in the work contexts must be evaluated constantly as values are changing and new things may become more important (Kristof-Brown et al., 2023). Items of Enjoyment and Traditional Values show lower internal consistencies than other constructs. In particular, evaluations of the bifactor model showed variations in standardized factor loadings and standard errors compared to other constructs and the classic CFA approach. The measurement of the underlying construct with the selected items may need further investigation as the dimensionality of work values can be more complex and heterogeneous than the 11 broader dimensions. This specifically accounts for Enjoyment with lower factor loadings, lower internal consistency, more distance in the MDS solution and a lack of higher correlations to individualistic work motives.

Furthermore, our goal was to provide evidence for convergent validity beyond basic human values (Consiglio et al., 2017; Albrecht et al., 2020). The correlations of the work values, to a great extent, implicate convergent validity. Nevertheless, the factorial validity of the used scales from the IEA-Short Form (Kanning, 2016) is low in this study. As a result, we appeal to be cautious when interpreting these relations. However, the correlations to the basic values provide unambiguous evidence for convergent validity, as expected by theoretical assumptions. Future research should also consider different variables in the nomological net, potentially assessing only specific variables per work value (comparable to our approach with Environmental Sustainability and environmental awareness).

We reported a bifactor model to control for a common variance factor with a good model fit to the data. Approximately 10% of the variance is attributable to this common variance factor. This is comparable to other common variance factors in value research (Lilleoja et al., 2016). However, the application of bifactor models is currently under debate concerning their accuracy in estimation and model specification (Mansolf and Reise, 2017; Eid et al., 2018; Rudnev, 2021). Hence, future research should consider a more accurate and comparable estimation of latent value factors by assessing proper approaches to control for common variance factors in value research.

As we collected all data from a single source at one time, one might argue that a common method bias is inherent to our data (Podsakoff et al., 2003). We acknowledge the discussion around this topic (Spector and Brannick, 2010). For potential improvements, we advocate that researchers survey relations of work values to other variables through multi-source and longitudinal data and across cultures. Here, the influence of previous organizational cultures on work value development should be considered. Variables like the length of employment, company size, and other socialization influences of previous organizations could be worth including as control variables above gender, age, and tenure in the current organization. The development of work values over time and the implications of work values in daily decision-making are promising research strings (as for value congruence and PO-Fit; Kristof-Brown et al., 2023). In the case of criterion validity, it would be beneficial to assess incremental validity over a period. We provided initial evidence for additional explained variance in the value congruence to the organization, but more comprehensive criteria must be assessed against the basic values (e.g., job performance, organizational citizenship behavior, or more narrowly defined attitudes toward diversity and organizational justice). Moreover, cross-cultural validations of the CWVS would be appropriate for evaluating measurement invariance.

Additional questionnaires are needed to assess the extent to which work value-based behavior can be relevant for organizational practice and personnel development. Correspondingly, a questionnaire to assess the perceived organizational culture based on the work value dimensions can deliver relevant insights into discrepancies between the importance and the lived reality of one’s personal work values. This would enable teams or employees to unfold aspects that oppose personal values and, therefore, cause strains.

## 6 Conclusion

Overall, our data promote the application of the theory of basic human values to the German work context. We used a genetic algorithm to derive a valid questionnaire, which supports strict measurement invariance between gender and age groups. The analysis of convergent, discriminant, and congruent incremental validity, in addition to the MDS results, strongly corroborates our hypothesized relations. The study results in a promising questionnaire for broader work value assessments with practical relevance.

## Data availability statement

The raw data supporting the conclusions of this article will be made available by the authors, without undue reservation by request.

## Ethics statement

The studies involving humans were approved by MSB Medical School Berlin Ethical Board. The studies were conducted in accordance with the local legislation and institutional requirements. The participants provided their written informed consent to participate in this study.

## Author contributions

JS: Conceptualization, Data curation, Formal analysis, Methodology, Writing – original draft. CS: Data curation, Resources, Supervision, Writing – review & editing. KH: Resources, Supervision, Writing – review & editing. TL: Conceptualization, Data curation, Formal analysis, Methodology, Project administration, Supervision, Validation, Writing – review & editing.

## Footnotes

^1^https://aspredicted.org/blind.php?x=ZMJ_9TJ

^2^Migration background is defined as having at least one parent who is a first-generation immigrant.

^3^Three attention check items; additional data collection to compensate for attention failure; “Contact Researcher”-option; an accurate estimate of needed time; pilot test with 10–30 participants; analysis for carelessness in answers; asking participants if they were attentive and offered data deletion option; an explanation for careful data entries, which participants had to check off.

^4^When selecting the interviewees, care was taken to ensure the greatest possible diversity in terms of age, professional position, educational background, and gender.

^5^Work value “Safety”: corporate social benefits were mentioned several times as important; work value “Traditional Values”: implementation of religious beliefs in working life was perceived as irritating by some respondents. As the aspect of religiosity is losing importance in everyday life in Germany (Hodapp and Zwingmann, 2019), the item was removed.

^6^We tested three- and four-item versions per dimension. As both had similar relations in the nomological network, we decided to use a three-item version. The three-item version seems rather stable except for the scales of Safety, Enjoyment, and Helping and Supporting. Here, multiple solutions were distinguishable. This will be addressed in the discussion.

^7^Deviation of the overall model from the perfect fit (*χ*^2^-test), Quotient of the test statistic (good: *χ*^2^/df ≤ 2; acceptable: *χ*^2^/df ≤ 3), CFI (good: ≥0.95; acceptable: ≥0.90), SRMR (good: ≤0.05; acceptable: ≤0.10) (Hu and Bentler, 1999), and RMSEA (good: ≤0.05; acceptable: ≤0.08) (Steiger, 1990).

## References

[ref1] Aczel B. Szaszi B. Sarafoglou A. Kekecs Z. Kucharský Š. Benjamin D.. (2020). A consensus-based transparency checklist. Nat. Hum. Behav. 4, 4–6. doi: 10.1038/s41562-019-0772-6, PMID: 31792401 PMC8324470

[ref2] Aguinis H. Villamor I. Ramani R. S. (2021). MTurk research: review and recommendations. J. Manag. 47, 823–837. doi: 10.1177/0149206320969787

[ref3] Albrecht S. Marty A. Brandon-Jones N. J. (2020). Measuring values at work: extending existing frameworks to the context of work. J. Career Assess. 28, 531–550. doi: 10.1177/1069072720901604

[ref4] Algner M. Lorenz T. (2022). You’re prettier when you smile: construction and validation of a questionnaire to assess microaggressions against women in the workplace. Front. Psychol. 13:809862. doi: 10.3389/fpsyg.2022.809862, PMID: 35369207 PMC8966652

[ref5] Alwin D. F. Krosnick J. A. (1985). The measurement of values in surveys: a comparison of ratings and rankings. Public Opin. Q. 49, 535–552. doi: 10.1086/268949

[ref6] Anglim J. Molloy K. Dunlop P. D. Albrecht S. L. Lievens F. Marty A. (2022). Values assessment for personnel selection: comparing job applicants to non-applicants. Eur. J. Work Organ. Psy. 31, 524–536. doi: 10.1080/1359432X.2021.2008911

[ref7] Anglim J. Sojo V. Ashford L. J. Newman A. Marty A. (2019). Predicting employee attitudes to workplace diversity from personality, values, and cognitive ability. J. Res. Pers. 83:103865. doi: 10.1016/j.jrp.2019.103865

[ref8] Arciniega L. González L. (2000). Development and validation of the work values scale EVAT 30. Int. J. Soc. Psychol. 15, 281–296. doi: 10.1174/021347400760259712

[ref9] Arieli S. Sagiv L. Roccas S. (2020). Values at work: the impact of personal values in organisations. Appl. Psychol. 69, 230–275. doi: 10.1111/apps.12181

[ref9001] Arthur W. Jr. Bell S. T. Villado A. J. Doverspike D. (2006). The use of person-organization fit in employment decision making: An assessment of its criterion-related validity. Journal of Applied Psychology, 91, 786–801. doi: 10.1037/0021-9010.91.4.786 16834506

[ref10] Avallone F. Farnese M. L. Pepe S. Vecchione M. (2010). The work values questionnaire (WVQ): revisiting Schwartz’s portrait values questionnaire (PVQ) for work contexts. Appl. Psychol. Bull., 59–76.

[ref11] Blum C. Roli A. (2003). Metaheuristics in combinatorial optimization: overview and conceptual comparison. ACM Comput. Surveys 35, 268–308. doi: 10.1145/937503.937505

[ref12] Boateng G. O. Neilands T. B. Frongillo E. A. Melgar-Quiñonez H. R. Young S. L. (2018). Best practices for developing and validating scales for health, social, and behavioral research: a primer. Front. Public Health 6:149. doi: 10.3389/fpubh.2018.00149, PMID: 29942800 PMC6004510

[ref13] Bojanowska A. Kaczmarek Ł. D. Urbanska B. Puchalska M. (2022). Acting on values: a novel intervention enhancing hedonic and eudaimonic well-being. J. Happiness Stud. 23, 3889–3908. doi: 10.1007/s10902-022-00585-4, PMID: 36213306 PMC9530432

[ref14] Borg I. Hertel G. Krumm S. Bilsky W. (2019). Work values and facet theory: from intercorrelations to individuals. Int. Stud. Manag. Organ. 49, 283–302. doi: 10.1080/00208825.2019.1623980

[ref15] Bühner M. Ziegler M. (2009). Statistik für Psychologen und Sozialwissenschaftler. Pearson Deutschland.

[ref16] Busque-Carrier M. Corff Y. L. Ratelle C. F. (2022). Development and validation of the integrative work values scale. Eur. Rev. Appl. Psychol. 72:100766. doi: 10.1016/j.erap.2022.100766

[ref17] Cable D. M. DeRue D. S. (2002). The convergent and discriminant validity of subjective fit perceptions. J. Appl. Psychol. 87, 875–884. doi: 10.1037/0021-9010.87.5.875, PMID: 12395812

[ref18] Cheung G. W. Rensvold R. B. (2002). Evaluating goodness-of-fit indexes for testing measurement invariance. Struct. Equ. Model. Multidiscip. J. 9, 233–255. doi: 10.1207/s15328007sem0902_5

[ref19] Consiglio C. Cenciotti R. Borgogni L. Alessandri G. Schwartz S. H. (2017). The WVal: a new measure of work values. J. Career Assess. 25, 405–422. doi: 10.1177/1069072716639691

[ref20] Curran P. G. (2016). Methods for the detection of carelessly invalid responses in survey data. J. Exp. Soc. Psychol. 66, 4–19. doi: 10.1016/j.jesp.2015.07.006

[ref21] Davidov E. Schmidt P. Schwartz S. H. (2008). Bringing values back in: the adequacy of the european social survey to measure values in 20 countries. Public Opin. Q. 72, 420–445. doi: 10.1093/poq/nfn035

[ref22] De Clercq S. Fontaine J. R. Anseel F. (2008). In search of a comprehensive value model for assessing supplementary person-organization fit. J. Psychol. 142, 277–302. doi: 10.3200/JRLP.142.3.277-302, PMID: 18589938

[ref23] Dörendahl J. Greiff S. (2020). Are the machines taking over? Benefits and challenges of using algorithms in (short) scale construction. Psychol. Assess. 36, 217–219. doi: 10.1027/1015-5759/a000597

[ref24] Dugard P. Todman J. Staines H. (2010). Approaching multivariate analysis: a practical introduction. Taylor & Francis.

[ref25] Eid M. Krumm S. Koch T. Schulze J. (2018). Bifactor models for predicting criteria by general and specific factors: problems of nonidentifiability and alternative5 solutions. J. Intelligence 6:42. doi: 10.3390/jintelligence6030042, PMID: 31162469 PMC6480823

[ref26] Etzel J. M. Nagy G. (2020). Challenging the multidimensional conception of perceived person-environment fit. Eur. J. Psychol. Assess. 37, 368–376. doi: 10.1027/1015-5759/a000622

[ref27] Fischer R. Smith P. B. (2006). Who cares about justice? The moderating effect of values on the link between organisational justice and work behaviour. Appl. Psychol. 55, 541–562. doi: 10.1111/j.1464-0597.2006.00243.x

[ref28] Flake J. K. Pek J. Hehman E. (2017). Construct validation in social and personality research: current practice and recommendations. Soc. Psychol. Personal. Sci. 8, 370–378. doi: 10.1177/1948550617693063

[ref29] Fuchs C. Diamantopoulos A. (2009). Using single-item measures for construct measurement in management research: conceptual issues and application guidelines. Die Betriebswirtschaft 69, 195–210.

[ref30] Gäde J. C. Schermelleh-Engel K. Brandt H. (2020). “Konfirmatorische Faktorenanalyse CFA” in Testtheorie und Fragebogenkonstruktion. eds. Moosbrugger H. Kelava A. (Heidelberg: Springer)

[ref31] Galán S. F. Mengshoel O. J. Pinter R. (2013). A novel mating approach for genetic algorithms. Evol. Comput. 21, 197–229. doi: 10.1162/EVCO_a_00067, PMID: 22264097

[ref32] Ghielen S. T. S. De Cooman R. Sels L. (2021). The interacting content and process of the employer brand: person-organization fit and employer brand clarity. Eur. J. Work Organ. Psy. 30, 292–304. doi: 10.1080/1359432X.2020.1761445

[ref33] Glazer S. Daniel S. C. Short K. M. (2004). A study of the relationship between organizational commitment and human values in four countries. Hum. Relat. 57, 323–345. doi: 10.1177/0018726704043271

[ref34] Gnambs T. (2013). Required sample size and power for sem. Available at: https://timo.gnambs.at/index.php/research/power-for-sem

[ref35] Gottfried J. Ježek S. Králová M. Řiháček T. (2022). Autocorrelation screening: a potentially efficient method for detecting repetitive response patterns in questionnaire data. Pract. Assess. Res. Eval. 27. doi: 10.7275/vyxb-gt24

[ref36] Hodapp B. Zwingmann C. (2019). Religiosity/spirituality and mental health: a Meta-analysis of studies from the German-speaking area. J. Relig. Health 58, 1970–1998. doi: 10.1007/s10943-019-00759-0, PMID: 30632002

[ref37] Holland J. H. (1992). Adaptation in natural and artificial systems: An introductory analysis with applications to biology, control, and artificial intelligence. Cambridge, Massachusetts: MIT press.

[ref38] Hout M. C. Papesh M. H. Goldinger S. D. (2013). Multidimensional scaling. Wiley Interdiscip. Rev. Cogn. Sci. 4, 93–103. doi: 10.1002/wcs.1203, PMID: 23359318 PMC3555222

[ref39] Hu L. T. Bentler P. M. (1999). Cutoff criteria for fit indexes in covariance structure analysis: conventional criteria versus new alternatives. Struct. Equ. Model. Multidiscip. J. 6, 1–55. doi: 10.1080/10705519909540118

[ref40] Hussey I. Hughes S. (2020). Hidden invalidity among 15 commonly used measures in social and personality psychology. Adv. Methods Pract. Psychol. Sci. 3, 166–184. doi: 10.1177/2515245919882903

[ref41] International Test Commission (2017). The ITC guidelines for translating and adapting. Int. J. Test. 18, 101–134. doi: 10.1080/15305058.2017.1398166

[ref42] ISSP Research Group (2017). International social survey programme: Work orientations IV - ISSP 2015

[ref43] Johnson M. K. (2001). Change in job values during the transition to adulthood. Work. Occup. 28, 315–345. doi: 10.1177/0730888401028003004

[ref44] Kanning U. P. (2016). IEA: Inventar zur Erfassung von Arbeitsmotiven: Manual Hogrefe.

[ref45] Kerber A. Schultze M. Müller S. Rühling R. M. Wright A. G. Spitzer C.. (2022). Development of a short and ICD-11 compatible measure for DSM-5 maladaptive personality traits using ant colony optimization algorithms. Assessment 29, 467–487. doi: 10.1177/1073191120971848, PMID: 33371717 PMC8866743

[ref46] Kline R. B. (2016). Principles and practice of structural equation modeling. Guilford Press.

[ref47] Kline R. B. (2019). Becoming a behavioral science researcher: A guide to producing research that matters. Guilford Press.

[ref48] Kooij D. T. De Lange A. H. Jansen P. G. Kanfer R. Dikkers J. S. (2011). Age and work-related motives: results of a meta-analysis. J. Organ. Behav. 32, 197–225. doi: 10.1002/job.665

[ref49] Kristof A. L. (1996). Person-organization fit: an integrative review of its conceptualizations, measurement, and implications. Pers. Psychol. 49, 1–49. doi: 10.1111/j.1744-6570.1996.tb01790.x

[ref50] Kristof-Brown A. L. Schneider B. Su R. (2023). Person-organization fit theory and research: conundrums, conclusions, and calls to action. Pers. Psychol. 76, 375–412. doi: 10.1111/peps.1258

[ref51] Kristof-Brown A. L. Zimmerman R. D. Johnson E. C. (2005). Consequences of individual ´s fit at work: a meta-analysis of person–job, person–organization, person–group, and person–supervisor fit. Pers. Psychol. 58, 281–342. doi: 10.1111/j.1744-6570.2005.00672.x

[ref52] Krumm S. Grube A. Hertel G. (2013). The Munster work value measure. J. Manag. Psychol. 28, 532–560. doi: 10.1108/JMP-07-2011-0023

[ref53] Kruskal J. B. (1964). Nonmetric multidimensional scaling: a numerical method. Psychometrika 29, 115–129. doi: 10.1007/BF02289694

[ref54] Lang J. W. B. Mussel P. Runge J. M. (2018). TBS-TK Rezension - Inventar zur Erfassung von Arbeitsmotiven (IEA). Zeitschrift Arbeits Organisationspsychol. 62, 161–163. doi: 10.1026/0932-4089/a000274

[ref55] Lechner C. Beierlein C. Davidov E. Schwartz S. H. (2022). Measuring the 4 higher-order values in Schwartz’s theory: a validation of a 17-item inventory. GESIS Zusammenstellung Sozialwissenschaftlicher Skalen Instrumente. doi: 10.31234/osf.io/xmh5v

[ref56] Leite W. L. Huang I.-C. Marcoulides G. A. (2008). Item selection for the development of short forms of scales using an ant colony optimization algorithm. Multivar. Behav. Res. 43, 411–431. doi: 10.1080/00273170802285743, PMID: 26741203

[ref57] Lilleoja L. Dobewall H. Aavik T. Strack M. Verkasalo M. (2016). Measurement equivalence of schwartz’s refined value structure across countries and modes of data collection: new evidence from Estonia, Finland, and Ethiopia. Personal. Individ. Differ. 102, 204–210. doi: 10.1016/j.paid.2016.07.009

[ref58] MacKenzie S. B. Podsakoff P. M. Podsakoff N. P. (2011). Construct measurement and validation procedures in MIS and behavioral research: integrating new and existing techniques. MIS Q. 35, 293–334. doi: 10.2307/23044045

[ref59] Maier M. Lakens D. (2022). Justify your alpha: a primer on two practical approaches. Adv. Methods Pract. Psychol. Sci. 5, 251524592210803–251524592210820. doi: 10.1177/25152459221080396

[ref60] Maio G. R. Pakizeh A. Cheung W.-Y. Rees K. J. (2009). Changing, priming, and acting on values: effects via motivational relations in a circular model. J. Pers. Soc. Psychol. 97, 699–715. doi: 10.1037/a0016420, PMID: 19785487

[ref61] Mansolf M. Reise S. P. (2017). When and why the second-order and bifactor models are distinguishable. Intelligence 61, 120–129. doi: 10.1016/j.intell.2017.01.012

[ref62] McCrae R. R. Costa P. T. (2003). Personality in adulthood: a five-factor theory perspective. New York: Guilford Press.

[ref63] Meredith W. (1993). Measurement invariance, factor analysis and factorial invariance. Psychometrika 58, 525–543. doi: 10.1007/BF02294825

[ref64] Moldzio T. Peiffer H. Wedemeyer P. S. Gentil A. (2021). Differentiated measurement of conscientiousness and emotional stability in an occupational context–greater effort or greater benefit? Eur. J. Work Organ. Psy. 30, 192–205. doi: 10.1080/1359432X.2020.1866066

[ref65] Mudge J. F. Baker L. F. Edge C. B. Houlahan J. E. (2012). Setting an optimal α that minimizes errors in null hypothesis significance tests. PLoS One 7:e32734. doi: 10.1371/journal.pone.0032734 22389720 PMC3289673

[ref66] Muthén L. K. Muthén B. O. (2017). Mplus user’s guide. 8th ed. Los Angeles: Muthén & Muthén.

[ref67] Nye C. D. (2022). Reviewer resources: confirmatory factor analysis. Organ. Res. Methods 26, 608–628. doi: 10.1177/10944281221120541

[ref68] Olaru G. Danner D. (2021). Developing cross-cultural short scales using ant colony optimization. Assessment 28, 199–210. doi: 10.1177/1073191120918026, PMID: 32418476

[ref69] Olaru G. Witthöft M. Wilhelm O. (2015). Methods matter: testing competing models for designing short-scale big-five assessments. J. Res. Pers. 59, 56–68. doi: 10.1016/j.jrp.2015.09.001

[ref70] Parks-Leduc L. Feldman G. Bardi A. (2015). Personality traits and personal values: a meta-analysis. Personal. Soc. Psychol. Rev. 19, 3–29. doi: 10.1177/1088868314538548 24963077

[ref71] Podsakoff P. M. MacKenzie S. B. Lee J.-Y. Podsakoff N. P. (2003). Common method biases in behavioral research: a critical review of the literature and recommended remedies. J. Appl. Psychol. 88, 879–903. doi: 10.1037/0021-9010.88.5.879, PMID: 14516251

[ref72] Porter C. O. L. H. Outlaw R. Gale J. P. Cho T. S. (2019). The use of online panel data in management research: a review and recommendations. J. Manag. 45, 319–344. doi: 10.1177/0149206318811569

[ref73] Potočnik K. Anderson N. R. Born M. Kleinmann M. Nikolaou I. (2021). Paving the way for research in recruitment and selection: recent developments, challenges and future opportunities. Eur. J. Work Organ. Psy. 30, 159–174. doi: 10.1080/1359432X.2021.1904898

[ref74] Pundt A. Kutzner J. Haberland K. Algner M. Lorenz T. (2022). You are simply not funny: development and validation of a scale to measure failed humor in leadership. Front. Psychol. 13:929988. doi: 10.3389/fpsyg.2022.929988, PMID: 35936334 PMC9355378

[ref75] Roccas S. Sagiv L. Schwartz S. H. Knafo A. (2002). The big five personality factors and personal values. Personal. Soc. Psychol. Bull. 28, 789–801. doi: 10.1177/0146167202289008

[ref76] Rönkkö M. Cho E. (2022). An updated guideline for assessing discriminant validity. Organ. Res. Methods 25, 6–14. doi: 10.1177/1094428120968614

[ref77] Ros M. Schwartz S. H. Surkiss S. (1999). Basic individual values, work values, and the meaning of work. Appl. Psychol. 48, 49–71. doi: 10.1111/j.1464-0597.1999.tb00048.x

[ref78] Rudnev M. (2021). Caveats of non-ipsatization of basic values: a review of issues and a simulation study. J. Res. Pers. 93:104118. doi: 10.1016/j.jrp.2021.104118

[ref79] Sackett P. R. Zhang C. Berry C. M. Lievens F. (2022). Revisiting meta-analytic estimates of validity in personnel selection: addressing systematic overcorrection for restriction of range. J. Appl. Psychol. 107, 2040–2068. doi: 10.1037/apl0000994, PMID: 34968080

[ref80] Sagiv L. Roccas S. Hazan O. (2004). Value pathways to well-being: healthy values, valued goal attainment, and environmental congruence. New Jersey: Wiley.

[ref81] Sagiv L. Schwartz S. H. (2022). Personal values across cultures. Annu. Rev. Psychol. 73, 517–546. doi: 10.1146/annurev-psych-020821-125100 34665670

[ref82] Sandy C. J. Gosling S. D. Koelkebeck T. (2014). Psychometric comparison of automated versus rational methods of scale abbreviation. J. Individ. Differ. 35, 221–235. doi: 10.1027/1614-0001/a000144

[ref83] Schleyer-Lindenmann A. Ittner H. Dauvier B. Piolat M. (2018). Die NEP-Skala – hinter den (deutschen) Kulissen des Umweltbewusstseins. Diagnostica 64, 156–167. doi: 10.1026/0012-1924/a000202

[ref84] Schmidt P. Bamberg S. Davidov E. Herrmann J. Schwartz S. H. (2007). Die Messung von Werten mit dem “Portraits Value Questionnaire”. Z. Sozialpsychol. 38, 261–275. doi: 10.1024/0044-3514.38.4.261

[ref85] Schroeders U. Schmidt C. Gnambs T. (2022). Detecting careless responding in survey data using stochastic gradient boosting. Educ. Psychol. Meas. 82, 29–56. doi: 10.1177/00131644211004708, PMID: 34992306 PMC8725053

[ref86] Schroeders U. Wilhelm O. Olaru G. (2016). Meta-heuristics in short scale construction: ant colony optimization and genetic algorithm. PLoS One 11:e0167110. doi: 10.1371/journal.pone.0167110, PMID: 27893845 PMC5125670

[ref87] Schultze M. (2017). Constructing subtests using ant colony optimization (doctoral dissertation). Freie Universität: Berlin

[ref88] Schupp J. Gerlitz J. (2014). Big five inventory-SOEP (BFI-S). Zusammenstellung Sozialwissenschaftlicher Items Skalen. doi: 10.6102/zis54

[ref89] Schwartz S. H. (1992). Universals in the content and structure of values: theoretical advances and empirical tests in 20 countries. Adv. Exp. Soc. Psychol. 25, 1–65. doi: 10.1016/S0065-2601(08)60281-6

[ref90] Schwartz S. H. (1994). Are there universal aspects in the structure and contents of human values? J. Soc. Issues 50, 19–45. doi: 10.1111/j.1540-4560.1994.tb01196.x

[ref91] Schwartz S. H. (2016). “Basic individual values: Sources and consequences”, in Handbook of value: perspectives from economics, neuroscience, philosophy, psychology and sociology. Eds. T. Brosch and D. Sander Oxford: Oxford University Press, 63–84.

[ref92] Schwartz S. H. (2021). A repository of Schwartz value scales with instructions and an introduction. Online Read. Psychol. Cult. 2:9. doi: 10.9707/2307-0919.1173

[ref93] Schwartz S. H. Boehnke K. (2004). Evaluating the structure of human values with confirmatory factor analysis. J. Res. Pers. 38, 230–255. doi: 10.1016/S0092-6566(03)00069-2

[ref94] Schwartz S. H. Cieciuch J. Vecchione M. Davidov E. Fischer R. Beierlein C.. (2012). Refining the theory of basic individual values. J. Pers. Soc. Psychol. 103, 663–688. doi: 10.1037/a0029393, PMID: 22823292

[ref95] Schwartz S. H. Melech G. Lehmann A. Burgess S. Harris M. Owens V. (2001). Extending the cross-cultural validity of the theory of basic human values with a different method of measurement. J. Cross-Cult. Psychol. 32, 519–542. doi: 10.1177/0022022101032005001

[ref96] Seifert K. H. Bergmann C. (1983). Deutschsprachige Adaptation des Work Values Inventory von Super: Ergebnisse bei Gymnasiasten und Berufstätigen. Psychol. Prax. 27, 160–172.

[ref97] Shaffer J. A. Postlethwaite B. E. (2012). A matter of context: a meta-analytic investigation of the relative validity of contextualized and noncontextualized personality measures. Pers. Psychol. 65, 445–494. doi: 10.1111/j.1744-6570.2012.01250.x

[ref98] Shaw M. Cloos L. J. Luong R. Elbaz S. Flake J. K. (2020). Measurement practices in large-scale replications: insights from many labs 2. Can. Psychol. 61, 289–298. doi: 10.1037/cap0000220

[ref99] Shi Z. Huang W. Liang Y. (2023). Work values and cultural background: a comparative analysis of work values of Chinese and British engineers in the UK. Front. Psychol. 14:1144557. doi: 10.3389/fpsyg.2023.1144557, PMID: 37275699 PMC10232762

[ref100] Simmons J. P. Nelson L. D. Simonsohn U. (2011). False-positive psychology: undisclosed flexibility in data collection and analysis allows presenting anything as significant. Psychol. Sci. 22, 1359–1366. doi: 10.1177/0956797611417632 22006061

[ref101] Spector P. E. Brannick M. T. (2010). Common method issues: an introduction to the feature topic in organizational research methods. Organ. Res. Methods 13, 403–406. doi: 10.1177/1094428110366303

[ref102] Steiger J. H. (1990). Structural model evaluation and modification: an interval estimation approach. Multivar. Behav. Res. 25, 173–180. doi: 10.1207/s15327906mbr2502_4, PMID: 26794479

[ref103] Stiglbauer B. Penz M. Batinic B. (2022). Work values across generations: development of the new work values scale (NWVS) and examination of generational differences. Front. Psychol. 13:1028072. doi: 10.3389/fpsyg.2022.1028072, PMID: 36420391 PMC9677943

[ref104] Straatmann T. Königschulte S. Hattrup K. Hamborg K.-C. (2020). Analysing mediating effects underlying the relationships between P-O fit, P-J fit, and organisational commitment. Int. J. Hum. Resour. Manag. 31, 1533–1559. doi: 10.1080/09585192.2017.1416652

[ref105] Sulistiobudi R. A. Hutabarat H. N. (2022). Adaptation of work values instrument in indonesian final year university students. Front. Psychol. 13:858688. doi: 10.3389/fpsyg.2022.858688, PMID: 35645909 PMC9133811

[ref106] Uggerslev K. L. Fassina N. E. Kraichy D. (2012). Recruiting through the stages: a meta-analytic test of predictors of applicant attraction at different stages of the recruiting process. Pers. Psychol. 65, 597–660. doi: 10.1111/j.1744-6570.2012.01254.x

[ref107] Wang Y. A. Rhemtulla M. (2021). Power analysis for parameter estimation in structural equation modeling: a discussion and tutorial. Adv. Methods Pract. Psychol. Sci. 4, 251524592091825–251524592091817. doi: 10.1177/2515245920918253

[ref108] Ward M. Meade A. W. (2023). Dealing with careless responding in survey data: prevention, identification, and recommended best practices. Annu. Rev. Psychol. 74, 577–596. doi: 10.1146/annurev-psych-040422-045007 35973734

[ref109] Yarkoni T. (2010). The abbreviation of personality, or how to measure 200 personality scales with 200 items. J. Res. Pers. 44, 180–198. doi: 10.1016/j.jrp.2010.01.002, PMID: 20419061 PMC2858332

[ref110] Zou G. Y. (2007). Toward using confidence intervals to compare correlations. Psychol. Methods 12, 399–413. doi: 10.1037/1082-989X.12.4.399, PMID: 18179351

