# Supplementary material for: Establishing circularity: development and validation of the circular work value scale (CWVS)
Source: Front Psychol. 2024 Apr 5;15:1296282. doi: 10.3389/fpsyg.2024.1296282 (PMC11026680; doi:10.3389/fpsyg.2024.1296282)
Supplement: Supplementary file 1 [file Data_Sheet_1.pdf]

1. **Transparency Checklist**

Establishing Circularity: Development and Validation of the Circular Work Value Scale (CWVS)

Transparency Report 1.0 (full, 36 items)

Corresponding author's email address: jannick.schneider@iao.fraunhofer.de

PREREGISTRATION SECTION

Prior to analyzing the complete data set, a time-stamped preregistration was posted in an independent, third-party registry for the data analysis plan. **Yes**

The manuscript includes a URL to all preregistrations that concern the present study. **Yes**

The study was preregistered… **before any data were collected**

The preregistration fully describes…

all inclusion and exclusion criteria for participation (e.g., English speakers who achieved a certain cutoff score in a language test). **NA**

all procedures for assigning participants to conditions. **NA**

all procedures for randomizing stimulus materials. **NA**

any procedures for ensuring that participants, experimenters, and data-analysts were kept naive (blinded) to potentially biasing information. **NA**

a rationale for the sample size used (e.g., an a priori power analysis). **Yes**

the measures of interest (e.g., friendliness). **Yes**

all operationalizations for the measures of interest (e.g., a questionnaire measuring friendliness). **Yes**

the data preprocessing plans (e.g., transformed, cleaned, normalized, smoothed). **Yes**

how missing data (e.g., dropouts) were planned to be handled. **NA**

the intended statistical analysis for each research question (this may require, for example, information about the sidedness of the tests, inference criteria, corrections for multiple testing, model selection criteria, prior distributions etc.). **Yes**

Comments about your Preregistration

No comments.

METHODS SECTION

The manuscript fully describes…

the rationale for the sample size used (e.g., an a priori power analysis). **Yes**

how participants were recruited. **Yes**

how participants were selected (e.g., eligibility criteria). **Yes**

what compensation was offered for participation. **Yes**

how participant dropout was handled (e.g., replaced, omitted, etc). **NA**

how participants were assigned to conditions. **NA**

how stimulus materials were randomized. **NA**

whether (and, if so, how) participants, experimenters, and data-analysts were kept naive to potentially biasing information. **NA**

the study design, procedures, and materials to allow independent replication. **Yes**

the measures of interest (e.g., friendliness). **Yes**

all operationalizations for the measures of interest (e.g., a questionnaire measuring friendliness). **Yes**

any changes to the preregistration (such as changes in eligibility criteria, group membership cutoffs, or experimental procedures). **NA**

Comments about your Methods section

No comments.

RESULTS AND DISCUSSION SECTION

The manuscript…

distinguishes explicitly between "confirmatory" (i.e., prespecified) and "exploratory" (i.e., not prespecified) analyses. **YES**

describes how violations of statistical assumptions were handled. **Yes**
justifies all statistical choices (e.g., including or excluding covariates; applying or not applying transformations; use of multi-level models vs. ANOVA). **Yes**
reports the sample size for each cell of the design. **NA**
reports how incomplete or missing data were handled. **NA**
presents protocols for data preprocessing (e.g., cleaning, discarding of cases and items, normalizing, smoothing, artifact correction). **Yes**

Comments about your Results and Discussion

No comments.

DATA, CODE, AND MATERIALS AVAILABILITY SECTION

The following have been made publicly available…

the (processed) data, on which the analyses of the manuscript were based. **No**
all code and software (that is not copyright protected). **No**
all instructions, stimuli, and test materials (that are not copyright protected). **NA**
Are the data properly archived (i.e., would a graduate student with relevant background knowledge be able to identify each variable and reproduce the analysis)? **NA**
The manuscript includes a statement concerning the availability and location of all research items, including data, materials, and code relevant to the study. **Yes**

Comments about your Data, Code, and Materials

No comments.

2. Initial item pool of the CWVS integrated in the survey

Tab A1: *Full 77 item scale with English translation and CFA results (n=853)*.

Value construct	German wording	English wording	Factor loadings	Standard errors
Authority	**1. Andere Menschen führen können**	**be able to lead other people**	.78	.05
	2.Kontrolle über begrenzte Ressourcen haben	have authority over limited resources	.65	.05
	**3.Bestimmen, wie Geld ausgegeben wird**	**determine how money is spent**	.69	.05
	4.Kontrolle über die Arbeitsaufgaben anderer Leute haben	have authority over other people's work programs	.81	.04
	**5.Entscheidungen darüber treffen können, wer welche Aufgaben übernimmt**	**make decisions about who does what**	.76	.05
	6.Durch meine berufliche Position bei anderen ein hohes Ansehen genießen	Be held in high reputation by others because of my job position	.72	.05
	7.Während der Arbeit Einfluss auf andere Personen haben können	Be able to influence other people while at work	.78	.04
Ambition	8.Meine Karriere vorantreiben	advance my career	.73	.06
	9.Die Ergebnisse der eigenen Anstrengung sehen	see the results of my own efforts.	.60	.04
	**10.In der Organisation als erfolgreich angesehen werden**	**be seen as successful in the organization**	.69	.04
	11.Befördert werden	get promoted	.69	.06
	**12.Ehrgeizig sein**	**be ambitious**	.77	.04
	**13.Leistung zeigen können**	**be able to show performance**	.73	.04
	14.Die Anerkennung meiner Fähigkeiten	having my skills recognized	.54	.04
Enjoyment	**15.Ausgleich zwischen beruflichen und erholsamen Tätigkeiten**	**Balance professional and recreational activities**	.59	.04
	16.Mich während der Arbeitszeit wohlfühlen	enjoy my time at work	.70	.03
	**17.Freude empfinden**	**have fun**	.69	.04
	**18.Dinge tun, die mir ein gutes Gefühl geben**	**doing things which make me feel good**	.69	.04
	19.Begeisterung für meine Arbeit haben	have enthusiasm for my work	.71	.04
	20.Die Arbeit mit meiner Freizeitgestaltung vereinbaren	balance work with my leisure time	.55	.05
	21.Arbeit und Privates in Einklang bringen	balance work and private life	.58	.04
Variety	**22.Abwechslungsreiche Aufgaben haben**	**do varied work**	.81	.04
	**23.Eine Vielfalt an Aufgaben bearbeiten**	**experience a wide variety of tasks**	.77	.04
	24.Abwechslung in meiner Arbeit haben	get a lot of variety in my work	.78	.04
	**25.Abwechslungsreiche Herausforderungen erleben**	**experience a wide variety of challenges**	.81	.04
	26.Mich nicht durch Wiederholungen langweilen	never be bored by repetition	.60	.04
	27.Bei der Arbeit Neues lernen	learn new things at work	.73	.04
	28.Verschiedene Fähigkeiten bei der Arbeit einsetzen	use different skills at work	.74	.03
Autonomy	29. Meine eigenen Entscheidungen bei der Arbeit treffen	make my own decisions	.79	.03
	**30. Entscheiden, wie ich meine Aufgaben erledige**	**be able to direct my own work**	.71	.03
	31.Eigenständig Dinge entwickeln und umsetzen	develop and implement things independently	.68	.04
	32.Die Möglichkeit haben, meine Aufgaben selbst zu bestimmen	have the opportunity to determine my own tasks.	.76	.03
	**33.Meine eigenen Prioritäten bei der Arbeit setzen**	**decide my own priorities at work**	.74	.03
	**34.Selbstständig und eigenverantwortlich handeln können**	**be able to act independently and on my own responsibility**	.73	.03
	35.Eine Tätigkeit ausführen, die unabhängiges Arbeiten ermöglicht	Perform a job that allows for independent work	.73	.03
Social Justice	**36.Mich für einen respektvollen Umgang in der Organisation einsetzen**	**to promote respectful behavior in the organization**	.71	.05
	**37.Zur Fairness in der Organisation beitragen**	**contribute to fairness in the organization**	.78	.05
	38.Zur sozialen Gerechtigkeit beitragen	continue to social justice	.84	.05
	39.In meiner Arbeit benachteiligte Menschen unterstützen	support people who are disadvantaged	.78	.04
	40Mit meiner Arbeit zur Verbesserung der Gesellschaft beitragen	contribute to the improve society	.74	.05
	**41.Mich für Chancengleichheit der Kolleg:innen in meinem Arbeitsumfeld einsetzen**	**to advocate for equal opportunities for colleagues in my work environment**	.80	.05
	42.In einer Organisation arbeiten, die aktiv gegen Mobbing und Diskriminierung am Arbeitsplatz vorgeht	work in an organization that actively combats bullying and discrimination in the workplace	.76	.05
Environmental Sustainability	**43.Die Umwelt schützen**	**protect the environment**	.90	.04
	44.Einen Beitrag zur ökologischen Nachhaltigkeit leisten	contribute to environmental sustainability	.91	.04
	**45.In einer Organisation arbeiten, die den Umweltschutz unterstützt**	**work in an organization that supports environmental protection**	.88	.04
	46.Meine Arbeit ökologisch nachhaltig gestalten	make my work environmentally sustainable	.90	.04
	**47.Umweltbewusst handeln**	**act in an environmentally conscious way**	.90	.04
	48.Mit Kolleg:innen zusammenarbeiten, die dem Umweltschutz eine hohe Bedeutung geben	work with colleagues who place a high priority on environmental protection.	.86	.04
	49. Natürliche Ressourcenverschwendung vermeiden	avoid wasting natural resources	.79	.05
Helping and Supporting	**50.Menschen helfen, mit denen ich in Kontakt komme**	**help the people I come in contact**	.79	.04
	51.Kolleg:innen bei der Arbeit unterstützen	support colleagues in their work.	.74	.04
	52.Harmonische Beziehungen zu Kolleg:innen aufbauen	build harmonious relationships with colleagues.	.65	.05
	**53.Anderen Menschen durch meine Arbeit helfen**	**do work which helps other people**	.72	.04
	**54.Das Leben der Menschen, denen ich bei der Arbeit begegne, verbessern**	**improve the lives of people I encounter at work**	.70	.05
	55.In einem Team arbeiten, in dem man sich aufeinander verlassen kann	work in a team where people can rely on each other	.65	.05
	56.Einen hilfsbereiten Umgang unter Kolleg:innen gewährleisten	ensure helpful interactions among colleagues	.72	.05

Rule Respecting	57.In einem Arbeitsumfeld mit klaren Regeln arbeiten	work in an orderly workplace	.73	.04
	58.In einem Team arbeiten, in dem alle Kolleg:innen sich an vereinbarte Prozessabläufe halten	work in a team in which all colleagues adhere to agreed process flows	.76	.04
	**59.In einem Team arbeiten, in dem wir alle die Richtlinien der Organisation unterstützen**	**work in a group where we all support the organization's policies**	.75	.04
	**60.An einem Arbeitsplatz arbeiten, an dem Regeln eingehalten werden**	**work in a workplace where rules are respected**	.78	.04
	**61.Mit Kolleg:innen zusammenarbeiten, die die Regeln auch dann einhalten, wenn niemand sie beobachtet**	**work with colleagues who respect rules even when no one else sees them**	.71	.04
	62.In einer Organisation arbeiten, in der Management-Vorgaben respektiert werden	work in an organization where management guidelines are respected	.70	.04
	63.Eine Arbeit ausführen, bei der ich mich an klaren Regeln und Richtlinien orientieren kann	do a job where I can follow clear rules and guidelines	.73	.04
Traditional Values	64.Sich nach überlieferten Gewohnheiten und Werten der Organisation richten	be guided by traditional customs and values of the organization	.74	.04
	**65.Im Einklang mit den Überzeugungen meiner Familie zu arbeiten**	**be able to work according to the values of my family**	.51	.05
	**66.Eine Arbeit verrichten, die mit meinen kulturellen Werten übereinstimmt**	**do work that is consistent with my cultural values**	.54	.05
	67.Eine Arbeit verrichten, die vom Großteil der Gesellschaft anerkannt wird	do a job which society would support	.71	.05
	68.Eine Arbeit verrichten, die traditionell gutgeheißen wird	do work which would be traditionally approved of	.70	.05
	69. Die Kultur der Organisation neuen Kolleg:innen vermitteln	communicate the culture of the organization to new colleagues	.72	.05
	**70.Die Traditionen meiner Organisation fortführen**	**carry on the traditions of my organization**	.75	.05
Safety	**71.Zur Sicherheit meiner Kolleg:innen beitragen**	**contribute to the safety of my colleagues**	.53	.08
	72.Meine persönliche Gesundheit nicht gefährden	not endanger my personal health	.61	.05
	**73.Die Sicherheit des Arbeitsplatzes maximieren**	**maximize job security**	.65	.06
	74.Mit einem gesicherten Einkommen rechnen können	be able to count on a secure income	.64	.06
	**75.Unterstützende Sozial- & Zusatzleistungen bereitgestellt durch die Organisation**	**supportive social & fringe benefits provided by the organization**	.52	.06
	76.Eine sichere berufliche Position haben	have a secure professional position	.71	.05
	77.Mit einer gesicherten beruflichen Zukunft rechnen können	be able to count on a secure professional future	.76	.04

Note: $\chi2$=7771.56, *df*=2794, *p*<.001, $\chi2$/*df*=2.78, CFI=.856, RMSEA=.046 [.045, .047], SRMR=.079; English translation through researchers (except for CWVS-items in bold)
